# Syntenin-1 is a promoter and prognostic marker of head and neck squamous cell carcinoma invasion and metastasis

**DOI:** 10.18632/oncotarget.13020

**Published:** 2016-11-02

**Authors:** Li Cui, Siliangyu Cheng, Xiaojun Liu, Diana Messadi, Yan Yang, Shen Hu

**Affiliations:** ^1^ University of California at Los Angeles, School of Dentistry, Los Angeles, CA 90095, USA; ^2^ University of California at Los Angeles, Jonsson Comprehensive Cancer Center, Los Angeles, CA 90095, USA; ^3^ University of California at Los Angeles, Department of Statistics, Los Angeles, CA 90095, USA; ^4^ Department of Stomatology, Zhongnan Hospital, Wuhan University, Wuhan 430071, China

**Keywords:** membrane proteins, metastasis, head and neck squamous cell carcinoma, syntenin-1

## Abstract

Metastasis represents a key factor associated with poor prognosis of head and neck squamous cell carcinoma (HNSC). However, the underlying molecular mechanisms remain largely unknown. In this study, our liquid chromatography with tandem mass spectrometry analysis revealed a number of significantly differentially expressed membrane/membrane-associated proteins between high invasive UM1 and low invasive UM2 cells. One of the identified membrane proteins, Syntenin-1, was remarkably up-regulated in HNSC tissues and cell lines when compared to the controls, and also over-expressed in recurrent HNSC and high invasive UM1 cells. Syntenin-1 over-expression was found to be significantly associated with lymph node metastasis and disease recurrence. HNSC patients with higher syntenin-1 expression had significantly poorer long term overall survival and similar results were found in many other types of cancers based on analysis of The Cancer Genome Atlas data. Finally, knockdown of syntenin-1 inhibited the proliferation, migration and invasion of HNSC cells, and opposite findings were observed when syntenin-1 was over-expressed. Collectively, our studies indicate that syntenin-1 promotes invasion and progression of HNSC. It may serve as a valuable biomarker for lymph node metastasis or a potential target for therapeutic intervention in HNSC.

## INTRODUCTION

Head and neck squamous cell carcinoma (HNSC) is the sixth most common type of cancer, accounting for approximately 2–3% of all malignancy worldwide [1, 2]. Despite the advances in surgery and radiochemotherapy for the disease treatment, the five-year survival rate of HNSC remains stagnant at about 50% in the past few decades [3, 4]. Metastasis has been demonstrated to be a key prognostic factor responsible for the poor clinical outcome of HNSC [5, 6]. Lymphatic spread is the major pathway for the dissemination of HNSC [7], and lung, liver and bone are the usual organs suffered from distant metastasis due to the hematogenous spread [8]. Understanding the molecular mechanisms that activate the metastasis process is an important strategy to improve the prognosis of HNSC.

Metastasis cascade represents a multi-step process, which includes physical detachment of cancer cells from the parental tumor, intravasation into blood or lymphatic vessels, survival in the circulation, extravasation, and subsequent proliferation in competent organs [9–11]. Membrane proteins especially cell surface molecules are essential for the metastasis process [12, 13]. Proteomics-based approach is an effective strategy for identification of novel metastasis-associated target proteins. We previously showed that the membrane proteomes were remarkably different between pancreatic ductal adenocarcinoma cells of primary and metastatic origin. Many of the identified proteins were regulators of cell-to-cell adhesion and tumor cell invasion [14]. Leth-Larsen et al. compared the membrane proteomes between high and low invasive breast cancer cell lines and identified 13 significantly deregulated membrane proteins. High expression of two identified molecules, ecto-5′-nucleotidase and integrin β1, in clinical samples were found to be closely correlated with poor clinical outcome which were measured as tumor spread or distant recurrence [15].

In this study, we first compared the membrane proteomes between high invasive UM1 and low invasive UM2 cells. A number of differentially expressed membrane and membrane-associated proteins, including syntenin-1 encoded by syndecan binding protein (SDCBP) gene, were identified. We then determined the role of syntenin-1 and its clinical significance in HNSC.

## RESULTS

### Differentially expressed membrane or membrane-associated proteins between UM1 and UM2 cells

**Figure 1:**
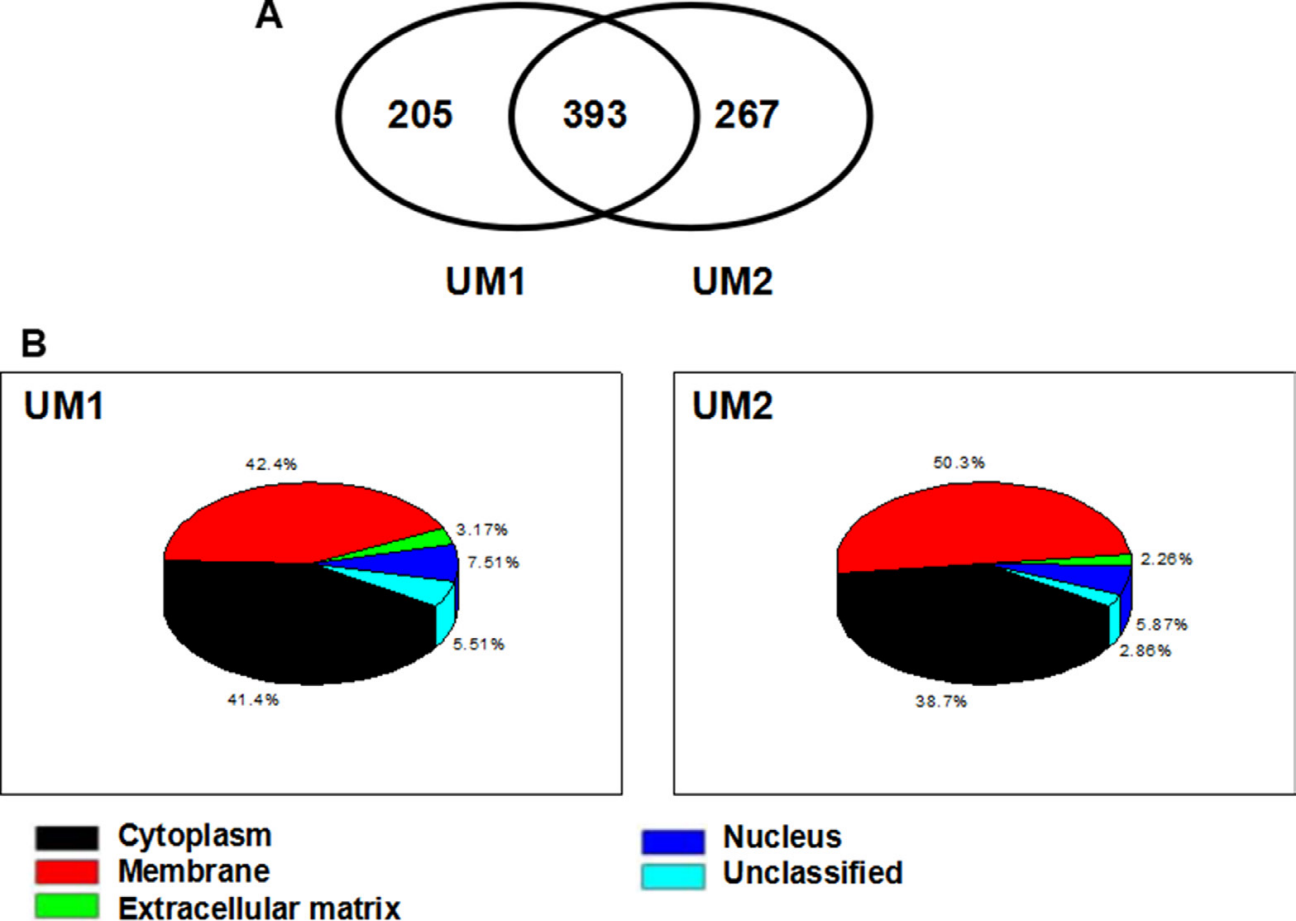
LC-MS/MS analysis and identification of membrane/membrane-associated proteins in UM1 and UM2 cells (**A**) The commonly and differentially expressed proteins between UM1 and UM2 cells; (**B**) Subcellular localization of the identified membrane and membrane-associated proteins in UM1 and UM2 cells.

In total, 598 and 660 membrane/membrane-associated proteins were identified from UM1 and UM2 cells, respectively, based on the liquid chromatography with tandem mass spectrometry (LC-MS/MS) analysis and database searching. Three hundred and ninety-three proteins were present in both cell lines but others were only found in either UM1 (*n* = 205) or in UM2 (*n* = 267) cells (Figure 1A). All the identified proteins and their relative information (access number, molecular weight, isoelectric point, number of unique peptides and number of total peptides) from the two cell lines were summarized in Supplementary Tables S1–S3. The subcellular localization of the membrane /membrane-associated proteins in UM1 and UM2 cells was shown in Figure 1B.

Representative membrane/membrane-associated proteins only found in UM1 or UM2 cells or in both cell lines were summarized in Tables 1–3, respectively. Membrane/membrane-associated proteins with metastasis-promoting function were prone to be found or had a higher expression level in UM1 cells versus UM2 cells. However, those proteins associated with adhesion property were more likely to be detected in UM2 cells.

**Table 1:** The representative membrane or membrane-associated proteins only identified in UM1 cells

Accession #	Protein name	Molecular weight (KDa)	PI	Number of unique peptides	Number of total peptides
P09525	Annexin A4	35.75	5.84	2	2
Q12791	Calcium-activated potassium channel subunit alpha-1	137.56	6.66	2	2
Q9H4A6	Golgi phosphoprotein 3	33.81	6.05	3	3
P04629	High affinity nerve growth factor receptor	84.17	5.99	2	2
P05362	Intercellular adhesion molecule 1	55.22	8.15	3	3
P18669	Phosphoglycerate mutase 1	28.67	6.75	4	7
P31948	Stress-induced-phosphoprotein 1	62.64	6.40	2	2
O00560	Syntenin-1	32.31	7.04	2	3

**Table 2:** The representative membrane/membrane-associated proteins only identified in UM2 cells

Accession #	Protein name	Molecular weight (KDa)	PI	Number of unique peptides	Number of total peptides
Q13740	CD166 antigen	62.26	5.71	6	11
6Q9UMD9	Collagen alpha-1(XVII) chain	150.42	8.89	4	4
P12111	Collagen alpha-3(VI) chain	340.81	6.15	2	2
P15924	Desmoplakin	331.77	6.44	4	5
P26006	Integrin alpha-3	113.51	6.13	2	3
P14923	Junction plakoglobin	81.74	5.75	4	6
O60716	Catenin delta-1	108.17	5.86	2	5
Q9UJZ1	Stomatin-like protein 2	35.69	5.37	6	38

**Table 3:** The representative membrane/membrane-associated proteins identified both in UM1 and UM2 cells

Accession #	Protein name	Molecular weight (KDa)	PI	Number of unique peptides (UM1/UM2)	Number of total peptides (UM1/UM2)
P07355	Annexin A2	38.47	7.56	25/24	330/227
P21964	Catechol O-methyltransferase	30.04	5.26	8/5	14/8
P23528	Cofilin-1	18.37	8.26	10/10	159/74
P15311	Ezrin	69.28	5.95	8/4	16/8
P09429	High mobility group protein B1	24.76	5.60	5/2	10/2
P17301	Integrin alpha-2	126.38	5.15	2/4	4/8
P05556	Integrin beta-1	86.19	5.27	4/8	14/22
Q9NQC3	Reticulon-4	129.93	4.42	8/8	183/29

### SDCBP is upregulated in HNSC and associated with poor prognosis

**Figure 2:**
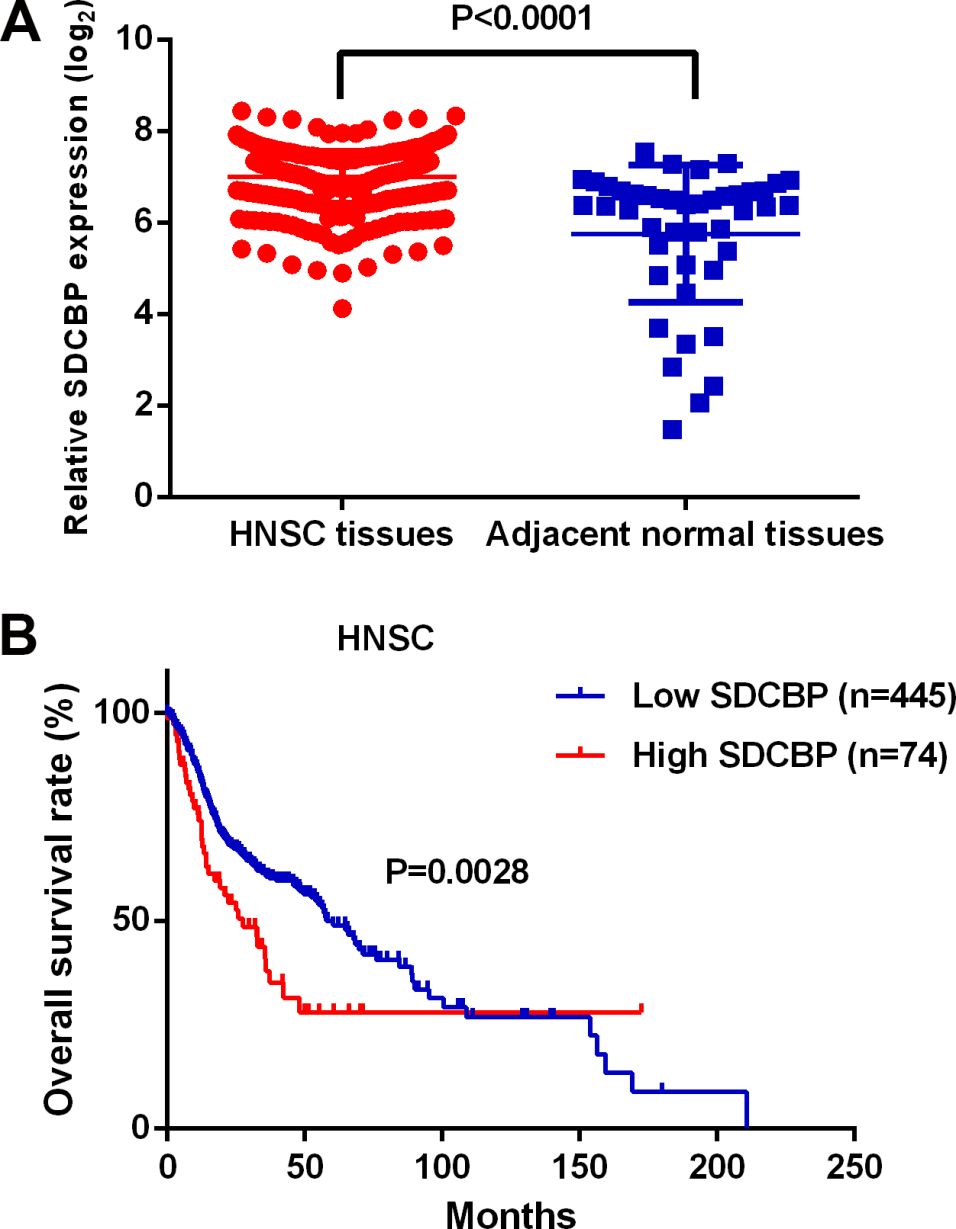
The expression level and clinical significance of *SDCBP* in HNSC (**A**) *SDCBP* was significantly up-regulated in HNSC tissues compared to the controls (*P* < 0.0001); (**B**) The HNSC patients in the high SDCBP expression group suffered poorer long term overall survival than those in the low *SDCBP* expression group (*P* = 0.0028).

**Figure 3:**
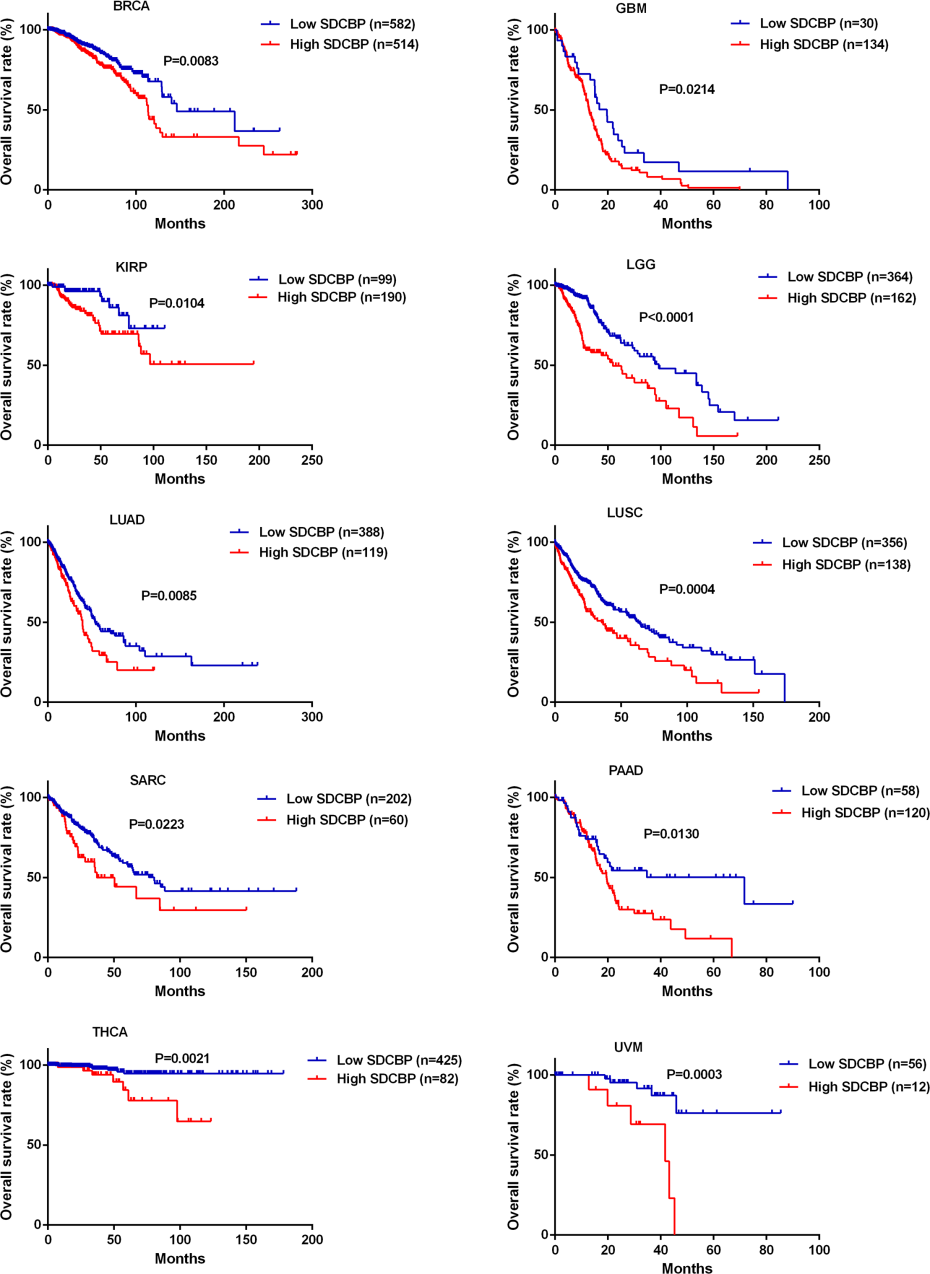
Higher *SDCBP* expression was associated with poorer overall survival in other types of cancers The cancer patients with higher *SDCBP* expression suffered remarkably shorter overall survival in a number of other cancers including BRCA (*P* = 0.0083), GBM (*P* = 0.0214), KIRP (*P* = 0.0104), LGG (*P* < 0.0001), LUAD (*P* = 0.0085), LUSC (*P* = 0.0004), SARC (*P* = 0.0223), PAAD (*P* = 0.0130), THCA (*P* = 0.0021) and UVM (*P* = 0.0003).

The expression level of SDCBP was significantly increased in HNSC tissues compared to the adjacent normal tissues (*P* < 0.0001, Figure 2A) and closely associated with lymph node metastasis (*P* = 0.0254) (Table 4). In addition, the HNSC patients in the high SDCBP expression group had remarkably shorter long-term overall survival (*P* = 0.0028, Figure 2B). Moreover, the cancer patients with higher expression of SDCBP had poorer long-term overall survival rates in many other types of cancers including breast cancer (BRCA, *P* = 0.0083), glioblastoma (GBM, *P* = 0.0214), kidney renal papillary cell carcinoma (KIRP, *P* = 0.0104), low grade glioma (LGG, *P* < 0.0001), lung adenocarcinoma (LUAD, *P* = 0.0085), lung squamous cell carcinoma (LUSC, *P* = 0.0004), sarcoma (SARC, *P* = 0.0223), prostate adenocarcinoma (PAAD, *P* = 0.0130), thyroid carcinoma (THCA, *P* = 0.0021) and uveal melanoma (UVM, *P* = 0.0003) (Figure 3).

**Table 4:** The association between *SDCBP* expression level and clinicopathological parameters of HNSC

Clinicopathological features	*SDCBP* expression		*P*
	Low	High	
**Age**	60.82 ± 11.84	61.22 ± 12.38	0.7937
**Gender**			
Female	111	27	0.0374
Male	334	47	
**T stage**			0.2549
T1-T2	164	22	
T3-T4	268	49	
**Lymph node metastasis**			0.0254
No	217	26	
Yes	209	45	
**Distant metastasis**			0.8478
M0	420	68	
M1	5	1	
Stage			0.1649
I-II	106	12	
III-IV	328	59	
**Angiolymphatic invasion**			0.0917
No	196	26	
Yes	103	23	
**Extracapsular spread**			0.1330
No	216	30	
Yes	90	20	
**Surgery margin status**			0.6062
Negative	297	51	
Positive	51	10	
Close	40	10	
**HPV 16 status**			0.2219
Negative	55	15	
Positive	36	5	

### Syntenin-1 is over-expressed in HNSC cell lines and tissues

**Figure 4:**
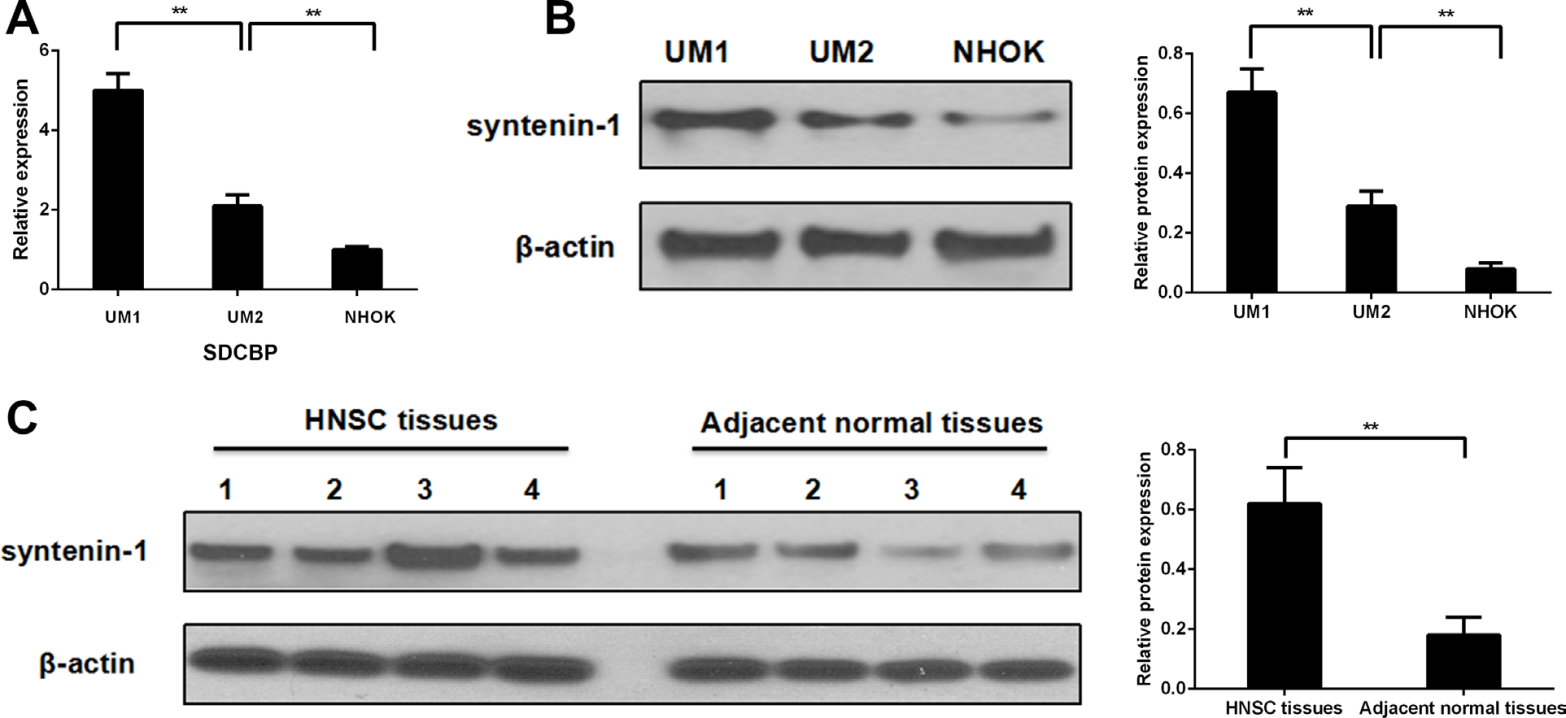
Syntenin-1 was overexpressed in HNSC cell lines and tissues (**A–B**) Syntenin-1 mRNA and protein levels were overexpressed significantly in both UM1 and UM2 cells compared to NHOKs (**P < 0.01). In addition, the expression level of syntenin-1 was significantly over-expressed in high invasive UM1 cells compared to low invasive UM2 cells (**P < 0.01). (**C**) The expression level of syntenin-1 was significantly overexpressed in HNSC tissues in comparison with the adjacent normal tissues (**P < 0.01).

Real-time PCR and Western blotting both showed that syntenin-1 was significantly over-expressed in both UM1 and UM2 cells when compared to NHOKs (*P* < 0.01) (Figure 4A). In addition, syntenin-1 was significantly upregulated in high invasive UM1 cells versus low invasive UM2 cells (*P* < 0.01) (Figure 4B).

**Figure 5:**
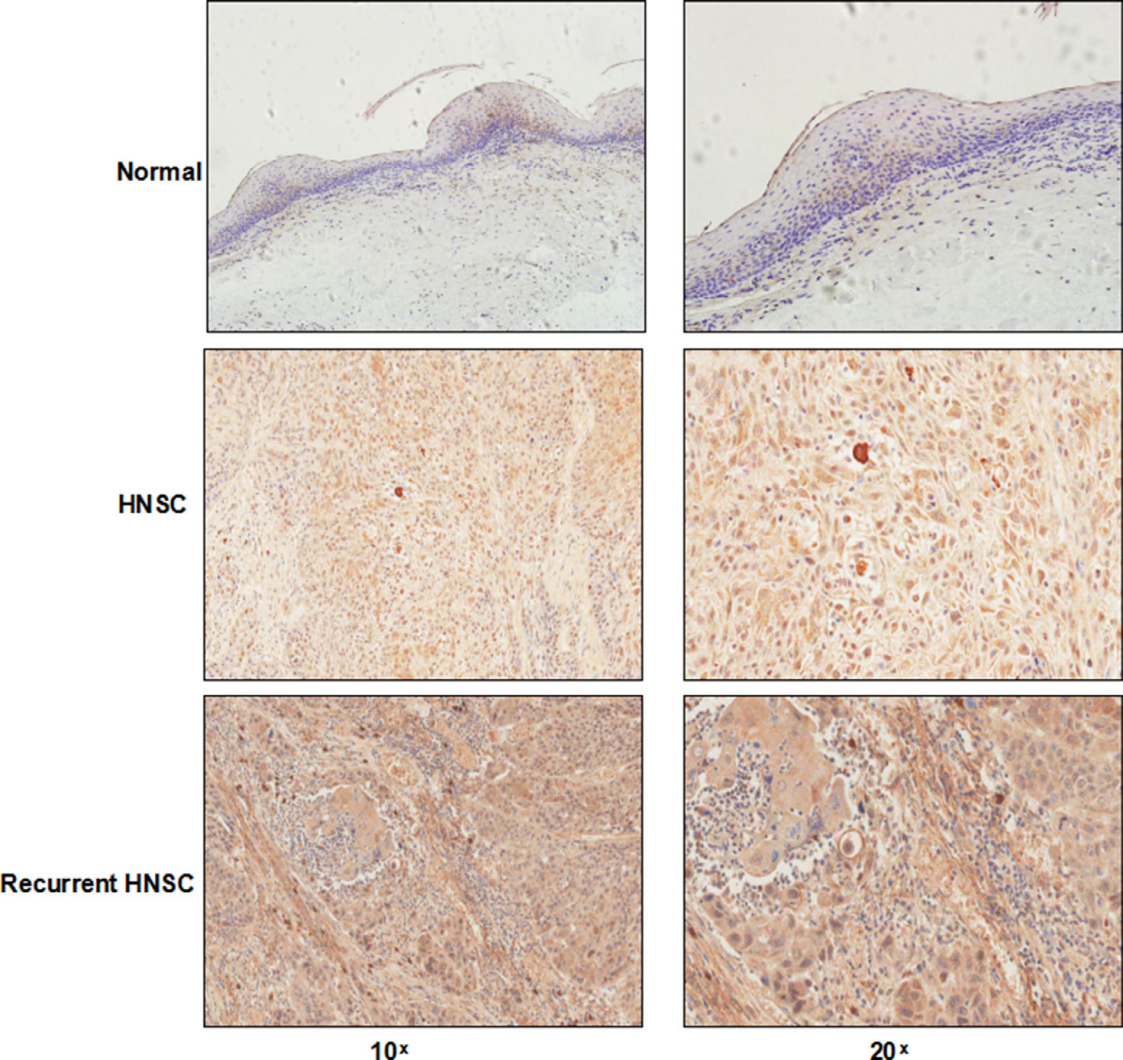
IHC analysis of syntenin-1 in HNSC Syntenin-1 was upregulated in HNSC while barely detected in normal tissues. The staining intensity of syntenin-1 was also higher in recurrent HNSC compared with non-recurrent HNSC.

The expression level of syntenin-1 was significantly upregulated in HNSC compared to adjacent normal tissues (*P* < 0.01) (Figure 4C). For the IHC analysis, the positive staining of cells was identified as bright yellow, yellow or brown-yellow granules. As shown in Figure 5, syntenin-1 was upregulated in HNSC but barely detected in normal tissues. In addition, the staining intensity of syntenin-1 was higher in recurrent HNSCs (Figure 5), and HNSC patients with higher IHC scores were significantly associated with lymph node metastasis (*P* = 0.0006) and disease recurrence (*P* = 0.0182) (Table 5).

**Table 5:** The association between syntenin-1 immunostaining and clinicopathological parameters of HNSC

Clinicopathological feature	Number	Staining score	*P*
**Age**			0.6369
<60	58	5.41 ± 2.11	
≥60	16	5.68 ± 1.72	
**Gender**			0.8422
Female	27	5.41 ± 1.99	
Male	47	5.51 ± 2.06	
**T stage**			0.2537
T1-T2	51	5.29 ± 2.05	
T3-T4	23	5.87 ± 1.95	
**Lymph node metastasis**			0.0006
No	39	4.73 ± 1.99	
Yes	35	6.30 ± 1.73	
**Recurrence**			0.0182
No	54	5.14 ± 2.05	
Yes	20	6.38 ± 1.67	
**Stage**			0.0670
I-II	25	4.87 ± 1.67	
III-IV	49	5.78 ± 2.14	
**Differentiation**			0.1383
Well	35	4.99 ± 2.16	
Moderate	23	5.76 ± 1.80	
Poor	16	6.10 ± 1.87	

### Down-regulation of syntenin-1 inhibits the proliferation, migration and invasion capacity of HNSC cells

**Figure 6:**
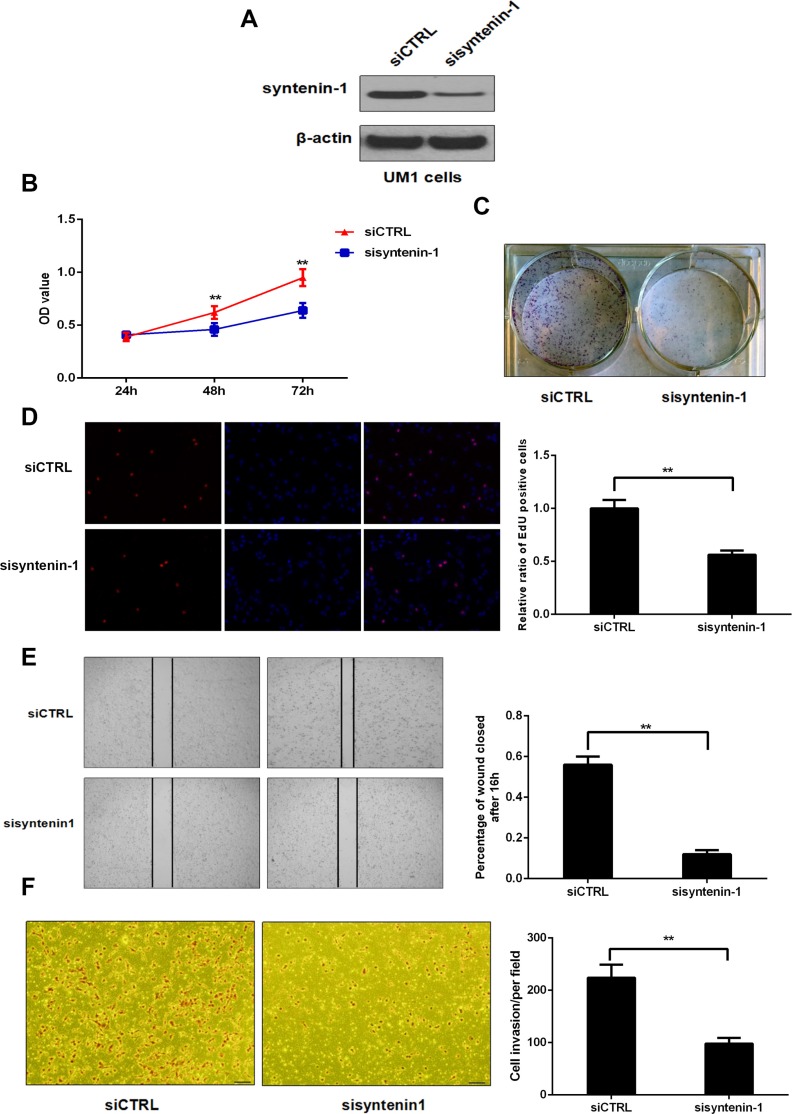
Inhibition of syntenin-1 suppressed the proliferation, migration and invasion capacity of UM1 cells (**A**) Syntenin-1 was significantly inhibited following sisyntenin-1 transfection. (**B**) The OD values were lower in syntenin-1 knockdown cells compared to the controls (**P < 0.01). (**C**) The number of colonies was significantly lower for syntenin-1 knockdown cells versus the controls. (**D**) The percentage of EdU-positive cells was lower for syntenin-1 knockdown cells (**P < 0.01). (**E**) The migration capability of syntenin-1 knockdown cells was significantly inhibited (**P < 0.01). (**F**) The number of cancer cells that invaded through the membrane was remarkably reduced following syntenin-1 knockdown (**P < 0.01).

Western blot analysis showed that the expression level of syntenin-1 was significantly suppressed in the UM1 cells transfected with syntenin-1 siRNA (sisyntenin-1) when compared to those transfected with scrambled siCTRL (Figure 6A). Cell proliferation was determined by an MTT assay in UM1 cells at 24 h, 48 h, and 72 h after siRNA transfection. UM1 cells with sisyntenin-1 transfection experienced a significant reduction in cell numbers when compared to the controls after 48 h and 72 h (*P* < 0.01) (Figure 6B). Meanwhile, UM1 cells with sisyntenin-1 transfection had suppressed growth capacity as indicated by the colony formation assay, with significantly fewer colonies formed than the cells transfected with siCTRL (Figure 6C). In addition, the percentage of EdU positive cells was lower in the cells with sisyntenin-1 transfection when compared to those with siCTRL transfection (*P* < 0.01) (Figure 6D).

Wound healing assay was performed to evaluate the effect of syntenin-1 inhibition on cell migration at 16 h after the cells reaching confluence. As shown in Figure 6E, in relative to siCTRL-transfected cells, the migration capability of cells transfected with sisyntenin-1 was significantly reduced. The wound gap between cell layers was significantly greater for the UM1 cells transfected with sisyntenin-1 when compared to those transfected with siCTRL (*P* < 0.01) (Figure 6E).

Matrigel invasion assay was used to measure the number of invaded UM1 cells after sisyntenin-1 or siCTRL transfection. The results showed that the cells transfected with sisyntenin-1 had a significant reduction in the number of cells crossing the transwell membrane when compared to those transfected with siCTRL (*P* < 0.01) (Figure 6F).

### Up-regulation of syntenin-1 promotes the proliferation, migration and invasion capacity of HNSC cells

**Figure 7:**
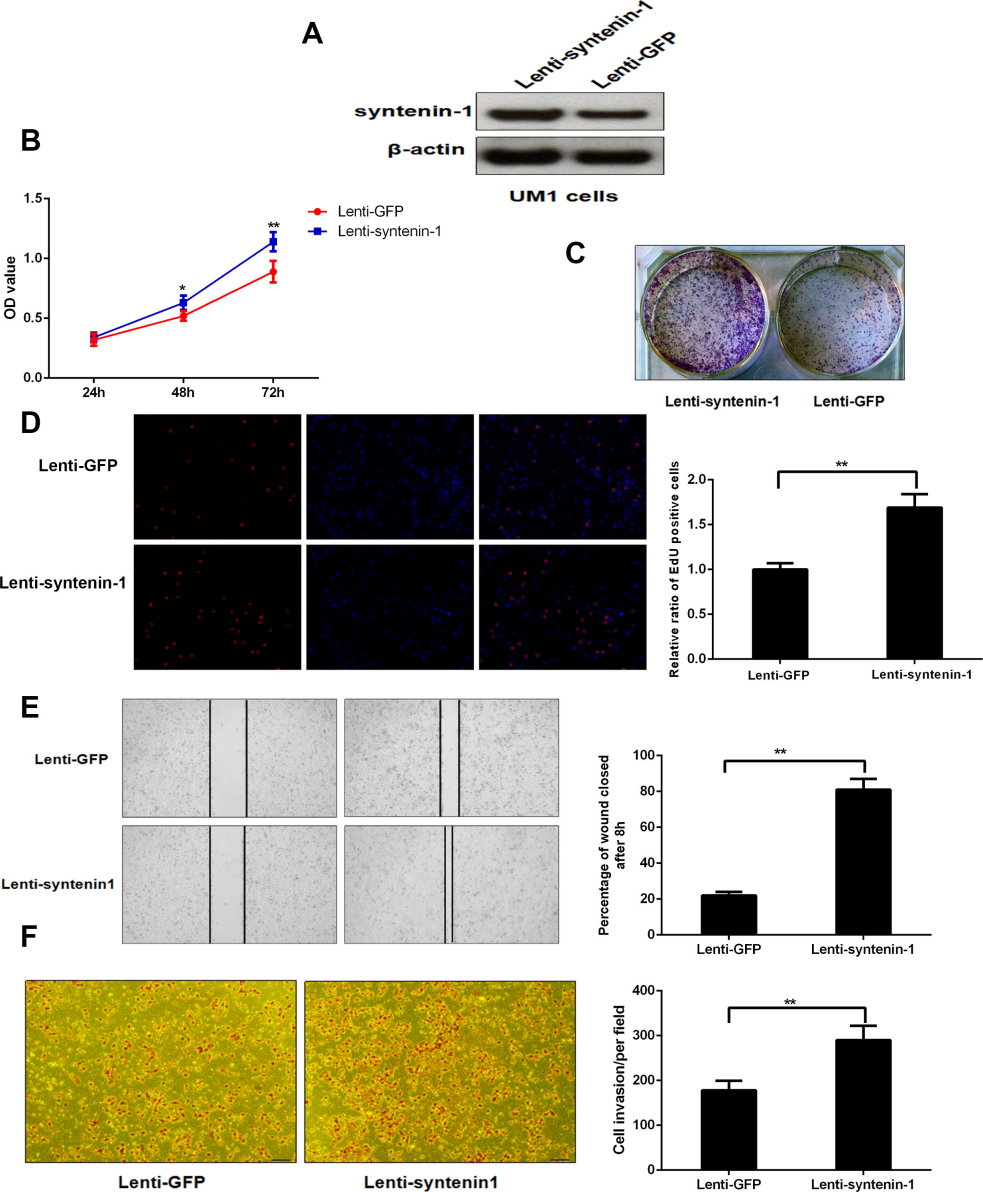
Syntenin-1 overexpression promoted the proliferation, migration and invasion capacity of UM1 cells (**A**) Syntenin-1 was significantly overexpressed following lenti-syntenin-1 infection. (**B**) The OD values were higher for syntenin-1 overexpressed cells when compared to the controls (*P < 0.05, **P < 0.01). (**C**) The number of colonies was significantly higher for syntenin-1 overexpressed cells versus the controls. (**D**) The percentage of EdU positive cells was higher for lenti-syntenin-1 treated cells (**P < 0.01). (**E**) The UM1 cells with syntenin-1 overexpression were more proficient than empty vector-transduced cells at closing an artificial wound (**P < 0.01). (**F**) The number of cancer cells that invaded through the membrane was remarkably increased following sisyntenin-1 up-regulation (**P < 0.01).

UM1 cells were successfully infected with lentiviral particles and the expression level of syntenin-1 protein was remarkably increased after lenti-syntenin-1 infection (Figure 7A). Similarly, the cell proliferation was assessed with the MTT, cell colony formation and EdU assays. The results indicated that the cells with syntenin-1 overexpression had higher OD values at 48 h (*P* < 0.05) and 72 h (*P* < 0.01) and stronger colony forming ability. In addition, syntenin-1 overexpression led to a higher percentage of EdU positive cells (*P* < 0.01) (Figure 7A–7D).

As shown in Figure 7E, our results indicated that UM1 cells with syntenin-1 overexpression were more proficient than empty vector-transduced cells at closing the artificial wound (*P* < 0.01). Syntenin-1 upregulation also significantly enhanced the invasive capacity of UM1 cells (*P* < 0.01).

## DISCUSSION

HNSC remains to be a significant public health problem and thus exploring the molecular mechanisms accounting for the development and progression of this malignancy is extremely crucial [16, 17]. In this study, we used an LC/MS-based approach to compare the membrane proteomes between highly and low invasive cancer cells and revealed a number of differentially expressed membrane/membrane-associated proteins. It should be noted that, although some proteins were only detected in UM1 cells, they may not be completely absent in UM2 cells, and vice versa. This is partially due to sensitivity limitation of LC-MS for analysis and identification of low abundant proteins and many membrane proteins are known to be expressed in low copy numbers [12, 14].

For the membrane/membrane-associated proteins only detected in UM1 cancer cells, many of them have a strong correlation with cancer invasion or metastasis. For instance, stress-induced-phosphoprotein 1 (STIP-1) is an adaptor protein that coordinates the functions of HSP70 and HSP90 in protein folding. Downregulation of STIP-1inhibited the migration and invasion capability of ovarian cancer cells [18]. Calcium-activated potassium channel subunit alpha-1 (KCNMA1) was overexpressed in malignant pleural mesothelioma (MPM), and KCNMA1 downregulation suppressed the migration of MPM cells [19]. On the other hand, many of the membrane or membrane associated proteins only found in UM2 cells have a functional role in cell-cell adhesion (e.g., desmoplakin, junction plakoglobin and stomatin-like protein 2), which connect the neighboring cells together and might accounting for the low metastatic capacity of UM2 cells. As for the differentially expressed proteins that were identified in both UM1 and UM2 cells, UM1 cells exhibited an over-expression of metastasis-related proteins but with under-expression of adhesion-related molecules in comparison to UM2 cells. For instance, the expression level of Reticulon-4 protein was about 6 times higher in UM1 cells than UM2 cells. In fact, previous study has shown that Reticulon-4 was overexpressed in human breast invasive ductal carcinoma and activated the epithelial-mesenchymal transition, indicating Reticulon-4 might be a metastasis-promoting molecule [20]. To the best of our knowledge, many of metastasis-associated proteins listed in Tables 1–3 were identified in HNSC for the first time. Further studies are warranted to investigate their role in the carcinogenesis of HNSC.

Our studies showed that syntenin-1 was overexpressed in HNSC cell lines as well as tissue samples. In addition, upregulation of syntenin-1 was associated with lymph node metastasis and disease recurrence. HNSC patients with higher syntenin-1 expression suffered poorer long-term overall survival. Moreover, syntenin-1 inhibition suppressed the proliferation, migration and invasion capacity of HNSC cells *in vitro*, and vice versa. These data demonstrated that syntenin-1 may function as an oncogenic molecule in HNSC and might serve as a potential target for therapeutic intervention. Syntenin-1, also known as melanoma differentiation associated gene-9 (mda-9), is a PDZ domain-containing adapter protein involving in various biological processes such as membrane trafficking, receptor clustering, cell adhesion, synaptic transmission, SOX4 activation, and exosome biogenesis [21]. Syntenin-1 deregulation has been reported in multiple primary cancers and its activity appears to be a driver of cancer progression. A number of signaling pathways including, but not limited to FAK, c-Src, p38-MAPK, AKT, NFkB, IGFBP2, EGFR, SPRR1B, and VEGFR may be mediated by syntenin-1 to promote tumorigenesis [22]. The role of syntenin-1 in cancer is most well studied in melanoma. The expression level of syntenin-1 was found to be overexpressed in human melanoma cell lines and patient derived tumor samples and associated with advanced stages of melanoma. Moreover, ectopic expression of syntenin-1 promoted the oncogenic activities of melanoma cells both *in vitro* and *in vivo*, and vice versa [23]. Das et al. showed that syntenin-1 induced the angiogenesis in melanoma by activating the expression of several angiogenesis-promoting factors including Src, FAK, AKT, HIF-1α and IGFBP-2, indicating syntenin-1 promoted the progression of melanoma [24]. Lastly but not the least, syntenin-1 was overexpressed in high metastatic breast cancer cell lines and tissues, and was significantly associated with the progression of breast cancer [25].

## MATERIALS AND METHODS

### Tissue samples and IHC analysis

Seventy-four formalin-fixed paraffin-embedded (FFPE) tissue specimens and the clinical information were obtained from the Department of Dentistry, Zhongnan Hospital, Wuhan University. This study was approved by the Institutional Research Ethics Committee at the Zhongnan Hospital, Wuhan University and the University of California, Los Angeles. Informed consent was obtained from all of patients or their relatives for the use of the tissues. For IHC analysis, FFPE tissue sections were deparaffinized by sequential washing with xylene, 100% ethanol, 95% ethanol, 80% ethanol and PBS. The endogenous peroxidase activity was quenched in methanol with 0.3% H_2_O_2_ for 5 min. The slides were blocked in PBS with 5% BSA for 30 min and then incubated overnight at 4°C with mouse antibody against human Syntenin-1 at a dilution of 1:100 (Santa Cruz Biotech, Santa Cruz, CA, USA). After rinsing in PBS, the sections were incubated with horseradish peroxidase (HRP)-conjugated sheep anti-mouse IgG (1:2000; GE Healthcare, Piscataway, NJ, USA) for 2 h at room temperature.

For the quantitative IHC analysis, the total staining score of syntenin-1 equals to the staining intensity (on a scale of 0–3: negative = 0, weak = 1, moderate = 2, and strong = 3) × the percentage of cells stained (on a scale of 0–3: 0 = zero, 1 = 1–25%, 2 = 26–50%, and 3 = 51–100%), resulting in a score range of 0–9. The evaluation was performed by two independent investigators.

### Analysis of SDCBP gene expression in tumor samples and its clinical significance

The clinical information and RNASeq V2 datasets of cancer patients were obtained from The Cancer Genome Atlas (TCGA) database (https://tcga-data.nci.nih.gov/tcga) to determine the clinical significance of syntennin-1 in cancers. Briefly the mRNA expression levels were log2-transformed and X tile software was used to find out the best cutoff point to divide the cancer patients into high/low SDCBP expression groups. Kaplan–Meier overall survival curves were generated for patients whose follow-up data were available. The log-rank test was used to analyze survival differences between the two groups.

### Cell culture

UM1 and UM2 oral cancer cell lines were cultured in the Dulbecco's modified eagle medium (DMEM) supplemented with 10% fetal bovine serum, penicillin (100 U/mL), and streptomycin (100 μg/mL). Normal human oral keratinocytes (NHOKs) were cultured in EpiLife media supplemented with the human keratinocyte growth supplement (Invitrogen, Carlsbad, CA, USA). The cells were maintained at 37°C, 5% CO_2_ in a humidified cell culture incubator and passaged when they reached 90–95% confluence.

### Sample preparation

The ProteoExtract Native Membrane Protein Extraction Kit (EMD Chemicals, Gibbstown, NJ) was used to isolate the membrane proteins from the UM 1 and UM 2 cells. Briefly, the cell pellet was washed three times in wash buffer, and then incubated with ice-cold Extract Buffer I under gentle agitation at 4°C for 10 min. Followed by centrifugation at 16,000g for 15 min at 4°C, the supernatant was discarded and 1 mL ice-cold Extract Buffer II was added to the pellet. This membrane protein extraction step was allowed for 30 min at 4°C under gentle agitation. Then the supernatant was collected after centrifugation at 16,000 g for 15 min 4°C.

### SDS-PAGE and proteolytic cleavage

2-D Quant Kit (GE Healthcare, Piscataway, NJ) was used to determine the total membrane protein concentration. From each cell line 20 μg of membrane protein was loaded into a 4–12% NuPAGE Bis-Tris gel (Invitrogen, Carlsbad, CA) for SDS-PAGE separation. The gel was stained with the Simply Blue staining solution (Invitrogen) to visualize the proteins. Each gel was then cut into 15 sections evenly and enzyme-grade trypsin (Promega, Madison, WI) was used to cleave the proteins into peptides in each section.

### Tandem MS and database searching

Liquid chromatography-tandem mass spectrometry (LC-MS/MS) analysis of peptides was performed using a NanoLC system (Eksigent Technologies, Dublin, CA) and a LTQ mass spectrometer (Thermo Fisher, Waltham, MA). Using an autosampler, 5 μL aliquots of the peptide digest derived from each gel slice were injected for analysis. Followed by concentrating and desalting on a C18 IntegraFrit Nano-Precolumn (New Objective, Woburn, MA), the peptides were separated using a C18 reversed-phase capillary column (New Objective). LC separation was performed at 400 nL/min with the following mobile phases: A, 5% acetonitrile/0.1%formic acid (v/v); B, 95% acetonitrile/0.1% formic acid (v/v). The chosen LC gradient was: from 5% to 15% B in 1 min, from 15% to 100% B in 40 min, and then maintained at 100%B for 15 min.

Database searches were performed using the X! Tandem search engine against the SwissProt protein sequence database. The search criteria were set with a mass accuracy of 0.4 Da and semi-style cleavage by trypsin. Positive identification of proteins was based on two or more unique peptides. The total number of peptides was used to estimate the abundance of a protein.

### siRNA knockdown of syntenin-1

UM1 cells were transfected with double-stranded siRNA using the RNAiMAX transfection regent (Invitrogen) according to the manufacturer's instruction. Validated siRNAs of syntenin-1 (sisyntenin-1, sc-42164) or scrambled control siRNAs (siCTRL, Santa Cruz Biotech) were mixed with the transfection reagent respectively and then added to the cell culture. After overnight incubation, the siRNAs were removed and the cells were further cultured in fresh media for 48 h before any additional experiments.

### Production of syntenin-1 recombinant lentiviral vectors

We cloned syntenin-1 into the pGCL-GFP vector and constructs were confirmed by sequencing. Recombinant lentiviral vectors and packaging vectors were then transfected into 293T cells. The supernatant liquor containing lentiviruses was harvested 72 hours after transfection. The lentiviruses were then purified by ultracentrifugation, and the titer of lentiviruses was determined. The empty vector was packaged as a negative control. The cancer cells were infected with the lentiviruses at a multiplicity of infection of 25.

### Real-time PCR

Total RNA was isolated from cancer cells using TRIzol (Takara, Dalian, China) according to the manufacturer's instructions. First-strand complementary DNA synthesis was performed using the SuperScript III Reverse Transcriptase (Invitrogen). The complementary DNA levels were amplified with Light Cycler 480â SYBR Green I MasterMix (Roche, Applied Science, Indianapolis, IN, USA) using the CFX96 Real-Time PCR detection system (Bio-Rad Laboratories Inc., Hercules, CA, USA). Gene expression was normalized against glyceraldehyde 3-phosphate dehydrogenase (GAPDH), and triplicate reactions were performed in three separate experiments.

### Western blotting

The protein samples were loaded and separated on a 4–12% Bis-Tris NuPAGE gel (Invitrogen) and transferred onto a nitrocellulose membrane using a Trans-blot SD semi-dry transfer cell (Bio-Rad). The membranes were blocked for 2 h at room temperature in TBST buffer containing 5% nonfat milk (Santa Cruz Biotech), and incubated with mouse antibody against human Syntenin-1 (1:500; Santa Cruz Biotech) overnight, followed by HRP-linked sheep anti-mouse IgG (1:5000; GE Healthcare). Signal detection was performed with the ECL-Plus Western blotting reagent kit (GE Healthcare).

### MTT assay

After 24 h of serum starvation, the cells were seeded into a 96-well plate at a density of 4000 cells/well. At the indicated time points, 20μL of MTT (Sigma-Aldrich, St. Louis, MO, USA) dissolved in PBS at 5 mg/ml was added to each culture well. Following by incubation for 4 h at 37°C, the supernatant was then discarded and the precipitate dissolved in 200 μl of dimethyl sulfoxide (DMSO, Sigma). The absorbance of each well was measured using a Synergy HT microplate reader (BioTek Instruments, Winooski, VT, USA) at 490 nm.

### 5-ethynyl-2′-deoxyuridine assay

The 5-ethynyl-2′-deoxyuridine (EdU) detection kit (Invitrogen) was used to evaluate cell proliferation. According to the manufacturer's instructions, the cells were treated with 10 μmol/L EdU for 4 h at 37°C and fixed with 3.7% formaldehyde for 15 min. After cells were washed with 3% BSA in PBS, they were treated with 0.5% Triton X-100 (Sigma-Aldrich) for 20 min and stained with 1 × Click-iT reaction cocktail for 30 minutes at room temperature. After PBS wash, Hoechst 33342 dye was used to stain the cell nucleus at room temperature for 30 min. Images were captured under a confocal laser scanning microscope (Olympus, Center Valley, PA). The assay was repeated in triplicate.

### Cell colony formation assay

The cells were seeded into a 6-well plate at a density of 1000 cells/well in 2 mL medium. After incubation at 37°C for 14 days, the cells were washed three times with PBS and stained with 0.5% crystal violet for 30 min.

### Wound healing assay

The migratory ability of cancer cells was assessed with a wound healing assay. Briefly, 5 × 10^5^ cells were seeded into a 6-well plate and grown until confluent. Subsequently, vertical scratch wounds were prepared using micropipette tips. For quantification, four random images per well were photographed at the beginning and after 8 h or 16 h, and the distance between the edges of the wound were calculated and analyzed by the NIH Image J software.

### Transwell Matrigel invasion assay

The invasion assays were performed with the Transwell Matrigel Invasion Chambers (BD Biosciences. Bedford, MA, USA). Following 24 h serum starvation, trypsinized cells (5 × 10^4^ cells /well) were resuspended in DMEM containing 0.1% FBS and added to upper chamber of transwell inserts. DMEM supplemented with 10% FBS was used in the lower chamber to act as a chemoattractant. After 24 h, cells that had migrated through the membrane were fixed and stained with the HEMA 3 staining kit (Fisher Scientific, Pittsburgh, PA, USA). The invaded cells in four random fields were counted and expressed as the average number of cells per field under light microscopy (Eclipse TE2000, Nikon, Tokyo, Japan).

### Statistical analysis

The data were expressed as the mean ± standard deviation, and analyzed by the independent samples *t*-test and one-way ANOVA using the MedCalc (MedCalc Software Inc, Ostend, Belgium). The optimum cutoff point of mRNA expression in the TCGA patient cohort was calculated using X-tile software. Briefly, the expression data of SDCBP in cancer patients and the related patient survival information, including survival time and status (alive or dead), were loaded into X-tile. The cancer patients were then categorized into two groups (high and low SDCBP expression group) by running the “Kaplan–Meier” program. Chi-square test was used to find out the association between the SDCBP mRNA expression level and clinicopathological parameter of HNSCs. Kaplan–Meier method in combination with log-rank test was performed for the survival analysis, and *P* values < 0.05 was considered to be statistically significant.

## CONCLUSIONS

Our study has revealed a number of novel target membrane proteins that are warranted for further investigation of their role in HNSC invasion and metastasis. One of the identified target proteins, syntenin-1, was found to be overexpressed in HNSC cancer cells and tissues and significantly associated with poor prognosis and lymph node metastasis of HNSC. We also found that down-regulation/up-regulation of syntenin-1 inhibited/promoted the proliferation, migration and invasion of HNSC cells. Collectively, all these findings indicate that syntenin-1 is a promoter of HNSC invasion and metastasis and may serve as a prognostic biomarker of HNSC.

## SUPPLEMENTARY MATERIALS








