# Supplementary material for: Syntenin-1 is a promoter and prognostic marker of head and neck squamous cell carcinoma invasion and metastasis
Source: Oncotarget. 2016 Nov 2;7(50):82634–47. doi: 10.18632/oncotarget.13020 (PMC5347720; doi:10.18632/oncotarget.13020)
Supplement: Supplementary file 1 [file oncotarget-07-82634-s001.pdf]

Syntenin-1 is a promoter and prognostic marker of head and neck squamous cell carcinoma invasion and metastasis

Supplementary Materials

Supplementary Table S1: Membrane and membrane-associated proteins identified in UM1 cells. See Supplementary_Table_S1

Supplementary Table S2: Membrane and membrane-associated proteins identified in UM2 cells. See Supplementary_Table_S2

Supplementary Table S3: Membrane and membrane-associated proteins identified both in UM1 and UM2 cells. See Supplementary_Table_S3
