# Supplementary material for: Syntenin-1 is a promoter and prognostic marker of head and neck squamous cell carcinoma invasion and metastasis
Source: Oncotarget. 2016 Nov 2;7(50):82634–47. doi: 10.18632/oncotarget.13020 (PMC5347720; doi:10.18632/oncotarget.13020)
Supplement: Supplementary file 2 [file oncotarget-07-82634-s002.docx]

Supplemental Table 1: Membrane and membrane-associated proteins identified in UM1 cells.

| Accession number | Protein name | Molecular weigth (KDa) | PI | Nubmers of total peptides | Numbers of unique peptides |
| --- | --- | --- | --- | --- | --- |
| Q9Y6N5 | Sulfide:quinone oxidoreductase, mitochondrial | 49.96 | 9.51 | 6 | 5 |
| Q9Y5I2 | Protocadherin alpha-10 | 102.88 | 5.04 | 2 | 2 |
| Q9Y4K4 | Mitogen-activated protein kinase kinase kinase kinase 5 | 95.04 | 7.56 | 3 | 3 |
| Q9Y4F5 | Protein KIAA0284 | 171.69 | 6.39 | 2 | 2 |
| Q9Y490 | Talin-1 | 269.77 | 5.77 | 13 | 9 |
| Q9Y485 | DmX-like protein 1 | 337.84 | 5.91 | 29 | 4 |
| Q9Y3U8 | 60S ribosomal protein L36 | 12.25 | 11.59 | 2 | 2 |
| Q9Y3T6 | R3H and coiled-coil domain-containing protein 1 | 53.26 | 5.93 | 2 | 2 |
| Q9Y3I0 | UPF0027 protein C22orf28 | 55.21 | 6.77 | 4 | 4 |
| Q9Y371 | Endophilin-B1 | 40.8 | 5.78 | 2 | 2 |
| Q9Y2B0 | Protein canopy homolog 2 | 20.65 | 4.81 | 3 | 2 |
| Q9Y216 | Myotubularin-related protein 7 | 75.83 | 5.94 | 3 | 2 |
| Q9UQ80 | Proliferation-associated protein 2G4 | 43.79 | 6.13 | 6 | 2 |
| Q9UPA5 | Protein bassoon | 416.47 | 7.28 | 2 | 2 |
| Q9UNL2 | Translocon-associated protein subunit gamma | 21.08 | 9.61 | 3 | 2 |
| Q9UKY7 | Protein CDV3 homolog | 27.33 | 6.06 | 2 | 2 |
| Q9UK61 | Uncharacterized protein C3orf63 | 189.03 | 5.55 | 3 | 2 |
| Q9UI30 | tRNA methyltransferase 112 homolog | 14.2 | 5.21 | 2 | 2 |
| Q9P2P6 | StAR-related lipid transfer protein 9 | 516.34 | 5.91 | 2 | 2 |
| Q9P0X4 | Voltage-dependent T-type calcium channel subunit alpha-1I | 245.1 | 6.09 | 2 | 2 |
| Q9NZW4 | Dentin sialophosphoprotein | 131.12 | 3.57 | 2 | 2 |
| Q9NZ08 | Endoplasmic reticulum aminopeptidase 1 | 107.23 | 6.02 | 3 | 2 |
| Q9NYQ6 | Cadherin EGF LAG seven-pass G-type receptor 1 | 329.49 | 5.59 | 2 | 2 |
| Q9NYC9 | Dynein heavy chain 9, axonemal | 511.87 | 5.64 | 2 | 2 |
| Q9NX47 | E3 ubiquitin-protein ligase MARCH5 | 31.23 | 9 | 4 | 2 |
| Q9NR09 | Baculoviral IAP repeat-containing protein 6 | 530.25 | 5.67 | 2 | 2 |
| Q9NQU5 | Serine/threonine-protein kinase PAK 6 | 74.87 | 9.57 | 2 | 2 |
| Q9NQG5 | Regulation of nuclear pre-mRNA domain-containing protein 1B | 36.9 | 5.72 | 2 | 2 |
| Q9HCH0 | Nck-associated protein 5-like | 138.01 | 8.45 | 2 | 2 |
| Q9HC84 | Mucin-5B | 596.34 | 6.2 | 2 | 2 |
| Q9HB71 | Calcyclin-binding protein | 26.21 | 8.28 | 4 | 2 |
| Q9H583 | HEAT repeat-containing protein 1 | 242.24 | 6.11 | 2 | 2 |
| Q9H4A6 | Golgi phosphoprotein 3 | 33.81 | 6.05 | 3 | 3 |
| \|Q9GZT3 | SRA stem-loop-interacting RNA-binding protein, mitochondrial | 12.35 | 10.26 | 3 | 2 |
| Q9BZL6 | Serine/threonine-protein kinase D2 | 96.75 | 6.39 | 2 | 2 |
| Q9BZ95 | Histone-lysine N-methyltransferase NSD3 | 161.61 | 8.57 | 2 | 2 |
| Q9BXW9 | Fanconi anemia group D2 protein | 164.13 | 5.58 | 2 | 2 |
| Q9BTT0 | Acidic leucine-rich nuclear phosphoprotein 32 family member E | 30.69 | 3.76 | 3 | 2 |
| Q9BSJ2 | Gamma-tubulin complex component 2 | 102.53 | 6.38 | 3 | 2 |
| Q9BQE3 | Tubulin alpha-1C chain | 49.9 | 4.96 | 13 | 9 |
| Q99873 | Protein arginine N-methyltransferase 1 | 41.52 | 5.24 | 3 | 2 |
| Q99717 | Mothers against decapentaplegic homolog 5 | 52.26 | 7.63 | 2 | 2 |
| Q96T58 | Msx2-interacting protein | 402.25 | 7.35 | 2 | 2 |
| Q96RY5 | Protein cramped-like | 134.72 | 8.08 | 2 | 2 |
| Q96PV0 | Ras GTPase-activating protein SynGAP | 148.28 | 9.12 | 2 | 2 |
| Q96JN8 | Neuralized-like protein 4 | 166.91 | 5.56 | 2 | 2 |
| Q96HR3 | Mediator of RNA polymerase II transcription subunit 30 | 20.28 | 8.45 | 2 | 2 |
| Q96AY4 | Tetratricopeptide repeat protein 28 | 270.88 | 6.42 | 2 | 2 |
| Q93074 | Mediator of RNA polymerase II transcription subunit 12 | 243.08 | 6.63 | 2 | 2 |
| Q92793 | CREB-binding protein | 265.35 | 8.83 | 2 | 2 |
| Q92688 | Acidic leucine-rich nuclear phosphoprotein 32 family member B. | 28.79 | 3.93 | 5 | 4 |
| Q8WZ42 | Titin | 3816.03 | 6.02 | 13 | 13 |
| Q8NFH8 | RalBP1-associated Eps domain-containing protein 2 | 71.53 | 7.56 | 2 | 2 |
| Q8ND04 | Protein SMG8 | 109.68 | 7.74 | 2 | 2 |
| Q8NCW5 | Apolipoprotein A-I-binding protein | 31.67 | 7.56 | 2 | 2 |
| Q8N5K1 | CDGSH iron sulfur domain-containing protein 2. | 15.28 | 9.66 | 5 | 3 |
| Q8N4S7 | Progestin and adipoQ receptor family member 4 | 29.13 | 9.26 | 2 | 2 |
| Q8N4C8 | Misshapen-like kinase 1 | 149.82 | 7.85 | 2 | 2 |
| Q8N3D4 | EH domain-binding protein 1-like protein 1 | 161.85 | 4.8 | 2 | 2 |
| Q8IY81 | Putative rRNA methyltransferase 3 | 96.56 | 8.53 | 3 | 3 |
| Q8IWZ3 | Ankyrin repeat and KH domain-containing protein 1 | 269.46 | 5.46 | 4 | 3 |
| Q8IVL1 | Neuron navigator 2 | 268.17 | 9.13 | 3 | 3 |
| Q7Z7H5 | Transmembrane emp24 domain-containing protein 4 | 25.94 | 8.41 | 2 | 2 |
| Q7Z6Z7 | E3 ubiquitin-protein ligase HUWE1 | 481.9 | 5.1 | 4 | 2 |
| Q7L2H7 | Eukaryotic translation initiation factor 3 subunit M | 42.5 | 5.41 | 2 | 2 |
| Q6IAA8 | RhoA activator C11orf59 | 17.74 | 5.01 | 7 | 5 |
| Q63ZY3 | KN motif and ankyrin repeat domain-containing protein 2 | 91.17 | 5.43 | 3 | 3 |
| Q5XKE5 | Keratin, type II cytoskeletal 79 | 57.84 | 6.75 | 3 | 2 |
| Q5VWG9 | Transcription initiation factor TFIID subunit 3 | 103.58 | 9.13 | 3 | 3 |
| Q5VV67 | Peroxisome proliferator-activated receptor gamma coactivator-related protein 1 | 177.54 | 6.11 | 2 | 2 |
| Q5VV63 | Attractin-like protein 1 | 147.35 | 7.12 | 2 | 2 |
| Q5T1M5 | FK506-binding protein 15 | 133.63 | 5.11 | 5 | 4 |
| Q5JSZ5 | Protein BAT2-like 1 | 242.97 | 8.55 | 2 | 2 |
| Q5JSL3 | Dedicator of cytokinesis protein 11 | 237.67 | 7.87 | 2 | 2 |
| Q5H9R7 | Serine/threonine-protein phosphatase 6 regulatory subunit 3 | 97.67 | 4.5 | 4 | 3 |
| Q53SF7 | Cordon-bleu protein-like 1 | 131.79 | 6.22 | 2 | 2 |
| Q4VC44 | FLYWCH-type zinc finger-containing protein 1 | 80.11 | 8.68 | 2 | 2 |
| Q16762 | Thiosulfate sulfurtransferase | 33.3 | 6.83 | 2 | 2 |
| Q16658 | Fascin; Singed-like protein | 54.4 | 6.81 | 6 | 3 |
| Q15911 | Zinc finger homeobox protein 3 | 404.42 | 5.82 | 2 | 2 |
| Q15691 | Microtubule-associated protein RP/EB family member 1 | 29.87 | 5.02 | 3 | 2 |
| Q15365 | Poly(rC)-binding protein 1 | 37.5 | 6.66 | 4 | 3 |
| Q15233 | Non-POU domain-containing octamer-binding protein | 54.23 | 9.01 | 2 | 2 |
| Q15058 | Kinesin-like protein KIF14 | 186.5 | 8.06 | 2 | 2 |
| Q14993 | Collagen alpha-1(XIX) chain | 112.5 | 8.41 | 2 | 2 |
| Q14766 | Latent-transforming growth factor beta-binding protein 1 | 184.38 | 5.59 | 2 | 2 |
| Q14643 | Inositol 1,4,5-trisphosphate receptor type 1 | 313.93 | 5.71 | 4 | 3 |
| Q14517 | Protocadherin Fat 1 | 503.88 | 4.84 | 2 | 2 |
| Q14498 | RNA-binding protein 39 | 59.25 | 10.1 | 3 | 2 |
| Q14247 | Src substrate cortactin | 61.59 | 5.24 | 4 | 2 |
| Q14152 | Eukaryotic translation initiation factor 3 subunit A | 166.44 | 6.39 | 6 | 5 |
| Q14103 | Heterogeneous nuclear ribonucleoprotein D0 | 38.3 | 7.6 | 3 | 2 |
| Q13765 | Nascent polypeptide-associated complex subunit alpha | 23.38 | 4.52 | 4 | 2 |
| Q13761 | Runt-related transcription factor 3 | 44.36 | 9.53 | 2 | 2 |
| Q13310 | Polyadenylate-binding protein 4 | 70.78 | 9.31 | 5 | 4 |
| Q13283 | Ras GTPase-activating protein-binding protein 1 | 52.03 | 5.36 | 3 | 2 |
| Q13136 | Liprin-alpha-1 | 135.78 | 5.91 | 2 | 2 |
| Q12791 | Calcium-activated potassium channel subunit alpha-1 | 137.56 | 6.66 | 2 | 2 |
| Q05519 | Serine/arginine-rich splicing factor 11 | 53.41 | 10.52 | 2 | 2 |
| Q04917 | 14-3-3 protein eta | 28.09 | 4.76 | 4 | 2 |
| Q04695 | Keratin, type I cytoskeletal 17 | 48.11 | 4.97 | 2 | 2 |
| Q04637 | Eukaryotic translation initiation factor 4 gamma 1 | 175.49 | 5.25 | 4 | 2 |

| Q01469 | Fatty acid-binding protein, epidermal | 15.03 | 6.82 | 2 | 2 |
| --- | --- | --- | --- | --- | --- |
| Q01130 | Splicing factor, arginine/serine-rich 2 | 25.34 | 11.86 | 3 | 2 |
| Q00G26 | Perilipin-5 | 50.79 | 5.08 | 2 | 2 |
| Q00975 | Voltage-dependent N-type calcium channel subunit alpha-1B | 262.5 | 8.78 | 2 | 2 |
| Q00688 | Peptidyl-prolyl cis-trans isomerase FKBP3 | 25.01 | 9.29 | 4 | 2 |
| Q00610 | Clathrin heavy chain 1 | 191.48 | 5.48 | 45 | 25 |
| P84103 | Splicing factor, arginine/serine-rich 3 | 19.33 | 11.64 | 5 | 3 |
| P83731 | 60S ribosomal protein L24 | 17.78 | 11.26 | 3 | 2 |
| P78347 | General transcription factor II-I | 112.29 | 6.09 | 4 | 3 |
| P68032 | Actin, alpha cardiac muscle 1 | 41.78 | 5.23 | 8 | 3 |
| P67775 | Serine/threonine-protein phosphatase 2A catalytic subunit alpha isoform | 35.59 | 5.3 | 2 | 2 |
| P63261 | Actin, cytoplasmic 2 | 41.78 | 5.31 | 332 | 25 |
| P62906 | 60S ribosomal protein L10a | 24.7 | 9.94 | 6 | 5 |
| P62899 | 60S ribosomal protein L31 | 14.46 | 10.54 | 6 | 4 |
| P62750 | 60S ribosomal protein L23a | 17.56 | 10.44 | 2 | 2 |
| P62316 | Small nuclear ribonucleoprotein Sm D2 | 13.4 | 9.92 | 4 | 2 |
| P61313 | 60S ribosomal protein L15 | 24.01 | 11.62 | 13 | 3 |
| P61158 | Actin-related protein 3 | 47.24 | 5.61 | 6 | 5 |
| P60842 | Eukaryotic initiation factor 4A-I | 46.02 | 5.32 | 20 | 6 |
| P60228 | Eukaryotic translation initiation factor 3 subunit E | 52.09 | 5.72 | 5 | 3 |
| P56537 | Eukaryotic translation initiation factor 6 | 26.6 | 4.56 | 8 | 4 |
| P52907 | F-actin-capping protein subunit alpha-1 | 32.79 | 5.45 | 4 | 2 |
| P51531 | Probable global transcription activator SNF2L2 | 181.28 | 6.76 | 3 | 3 |
| P51153 | Ras-related protein Rab-13 | 22.52 | 9.27 | 2 | 2 |
| P50993 | Sodium/potassium-transporting ATPase subunit alpha-2 | 111.79 | 5.43 | 3 | 3 |
| P50990 | T-complex protein 1 subunit theta | 59.49 | 5.41 | 8 | 5 |
| P49915 | GMP synthase [glutamine-hydrolyzing] | 76.58 | 6.43 | 2 | 2 |
| P49721 | Proteasome subunit beta type-2 | 22.84 | 6.52 | 5 | 2 |
| P49321 | Nuclear autoantigenic sperm protein | 85.11 | 4.26 | 2 | 2 |
| P48444 | Coatomer subunit delta | 57.08 | 5.89 | 4 | 2 |
| P46939 | Utrophin | 394.45 | 5.2 | 4 | 4 |
| P46778 | 60S ribosomal protein L21 | 18.43 | 10.49 | 5 | 3 |
| P46060 | Ran GTPase-activating protein 1 | 63.41 | 4.63 | 2 | 2 |
| P43487 | Ran-specific GTPase-activating protein | 23.18 | 5.19 | 3 | 2 |
| P42677 | 40S ribosomal protein S27 | 9.33 | 9.58 | 3 | 2 |
| P37108 | Signal recognition particle 14 kDa protein | 14.44 | 10.05 | 3 | 2 |
| P35908 | Keratin, type II cytoskeletal 2 epidermal | 65.43 | 8.07 | 8 | 5 |
| P35527 | Keratin, type I cytoskeletal 9 | 62.06 | 5.14 | 2 | 2 |
| P34897 | Serine hydroxymethyltransferase, mitochondrial | 52.26 | 8.11 | 9 | 6 |
| P31948 | Stress-induced-phosphoprotein 1 | 62.64 | 6.4 | 2 | 2 |
| P30450 | HLA class I histocompatibility antigen, A-26 alpha chain | 38.55 | 5.96 | 11 | 5 |
| P30443 | HLA class I histocompatibility antigen, A-1 alpha chain | 38.32 | 5.96 | 3 | 2 |
| P29992 | Guanine nucleotide-binding protein subunit alpha-11 | 42.12 | 5.51 | 2 | 2 |
| P29400 | Collagen alpha-5(IV) chain | 158.35 | 7.53 | 2 | 2 |
| P28066 | Proteasome subunit alpha type-5 | 26.41 | 4.74 | 4 | 2 |
| P27708 | CAD protein; Includes: Glutamine-dependent carbamoyl-phosphate synthase | 242.85 | 6.02 | 4 | 2 |
| P27635 | 60S ribosomal protein L10 | 24.47 | 10.11 | 9 | 3 |
| P27348 | 14-3-3 protein theta | 27.76 | 4.68 | 12 | 6 |
| P25789 | Proteasome subunit alpha type-4 | 29.48 | 7.58 | 3 | 3 |
| P25788 | Proteasome subunit alpha type-3 | 28.3 | 5.19 | 2 | 2 |
| P24534 | Elongation factor 1-beta | 24.63 | 4.5 | 3 | 2 |
| P23381 | Tryptophanyl-tRNA synthetase, cytoplasmic | 53.03 | 5.83 | 3 | 2 |
| P23246 | Splicing factor, proline- and glutamine-rich | 76.15 | 9.45 | 4 | 4 |
| P19961 | Alpha-amylase 2B | 55.9 | 6.49 | 6 | 4 |
| P19367 | Hexokinase-1 | 102.49 | 6.36 | 4 | 2 |
| P18669 | Phosphoglycerate mutase 1 | 28.67 | 6.75 | 7 | 4 |
| P18621 | 60S ribosomal protein L17 | 21.27 | 10.18 | 27 | 3 |
| P17987 | T-complex protein 1 subunit alpha | 60.34 | 5.8 | 9 | 5 |
| P16112 | Aggrecan core protein | 248.35 | 4.1 | 2 | 2 |
| P15502 | Elastin | 65.82 | 10.32 | 4 | 4 |
| P13674 | Prolyl 4-hydroxylase subunit alpha-1 | 59.11 | 5.69 | 5 | 4 |
| P13073 | Cytochrome c oxidase subunit 4 isoform 1, mitochondrial | 17.2 | 9.16 | 6 | 3 |
| P12235 | ADP/ATP translocase 1 | 32.93 | 9.78 | 5 | 3 |
| P12004 | Proliferating cell nuclear antigen | 28.77 | 4.57 | 3 | 2 |
| P09972 | Fructose-bisphosphate aldolase C | 39.32 | 6.46 | 4 | 2 |
| P09525 | Annexin A4 | 35.75 | 5.84 | 2 | 2 |
| P08708 | 40S ribosomal protein S17; | 15.42 | 9.85 | 3 | 2 |
| P07199 | Major centromere autoantigen B | 65.04 | 4.49 | 2 | 2 |
| P07195 | L-lactate dehydrogenase B chain | 36.51 | 5.72 | 3 | 2 |
| P05455 | Lupus La protein | 46.84 | 6.68 | 5 | 4 |
| P05387 | 60S acidic ribosomal protein P2 | 11.67 | 4.38 | 2 | 2 |
| P05362 | Intercellular adhesion molecule 1 | 55.22 | 8.15 | 3 | 3 |
| P04629 | High affinity nerve growth factor receptor | 84.17 | 5.99 | 2 | 2 |
| P02538 | Keratin, type II cytoskeletal 6A | 59.91 | 8.14 | 9 | 5 |
| P02533 | Keratin, type I cytoskeletal 14 | 51.56 | 5.09 | 4 | 3 |
| P02461 | Collagen alpha-1(III) chain | 95.29 | 9.36 | 2 | 2 |
| P01889 | HLA class I histocompatibility antigen, B-7 alpha chain | 37.89 | 5.57 | 2 | 2 |
| P01833 | Polymeric immunoglobulin receptor | 81.35 | 5.59 | 3 | 2 |
| P01111 | GTPase NRas | 20.9 | 5.01 | 2 | 2 |
| P01037 | Cystatin-SN | 14.32 | 6.92 | 23 | 2 |
| P00451 | Coagulation factor VIII | 264.73 | 6.97 | 2 | 2 |
| O95153 | Peripheral-type benzodiazepine receptor-associated protein 1 | 200.05 | 5.05 | 4 | 3 |
| O95104 | Splicing factor, arginine/serine-rich 15 | 125.87 | 9.58 | 2 | 2 |
| O94856 | Neurofascin | 147.5 | 6.15 | 2 | 2 |
| O76003 | Glutaredoxin-3 | 37.3 | 5.31 | 3 | 2 |
| O75636 | Ficolin-3 | 30.35 | 6.22 | 2 | 2 |
| O75607 | Nucleoplasmin-3 | 19.21 | 4.55 | 7 | 2 |
| O75323 | Protein NipSnap homolog 2 | 33.74 | 9.42 | 2 | 2 |
| O75128 | Protein cordon-bleu | 135.61 | 7.66 | 2 | 2 |
| O43795 | Myosin-Ib; Myosin I alpha | 131.99 | 9.43 | 10 | 6 |
| O43432 | Eukaryotic translation initiation factor 4 gamma 3 | 176.65 | 5.27 | 3 | 3 |
| O43324 | Eukaryotic translation elongation factor 1 epsilon-1 | 19.68 | 8.58 | 3 | 2 |
| O15460 | Prolyl 4-hydroxylase subunit alpha-2 | 58.57 | 5.43 | 3 | 2 |
| O15144 | Actin-related protein 2/3 complex subunit 2 | 34.33 | 6.84 | 6 | 4 |
| O14907 | Tax1-binding protein 3 | 13.6 | 8.02 | 4 | 2 |
| O14818 | Proteasome subunit alpha type-7 | 27.89 | 8.6 | 2 | 2 |
| O14686 | Histone-lysine N-methyltransferase MLL2 | 593.39 | 5.4 | 5 | 5 |
| O00560 | Syntenin-1 | 32.31 | 7.04 | 3 | 2 |
| O00303 | Eukaryotic translation initiation factor 3 subunit F | 37.43 | 5.24 | 4 | 2 |
| A8MW95 | Beclin-1-like protein 1 | 48.15 | 4.78 | 2 | 2 |
| A7XYQ1 | Sine oculis-binding protein homolog | 92.66 | 7.77 | 2 | 2 |

| A1A4S6 | Rho GTPase-activating protein 10 | 89.37 | 6.75 | 2 | 2 |
| --- | --- | --- | --- | --- | --- |
| A0AVT1 | Ubiquitin-like modifier-activating enzyme 6 | 117.97 | 5.76 | 2 | 2 |
