# Supplementary material for: Syntenin-1 is a promoter and prognostic marker of head and neck squamous cell carcinoma invasion and metastasis
Source: Oncotarget. 2016 Nov 2;7(50):82634–47. doi: 10.18632/oncotarget.13020 (PMC5347720; doi:10.18632/oncotarget.13020)
Supplement: Supplementary file 3 [file oncotarget-07-82634-s003.docx]

Supplemental Table 2: Membrane and membrane-associated proteins identified in UM2 cells.

| Accession number | Protein name Molecular weight (KDa) | PI | Nubmers of total peptides | Nubmers of unique peptides |
| --- | --- | --- | --- | --- |
| A2RRP1 | Neuroblastoma-amplified sequence 268.57 | 5.65 | 2 | 2 |
| A2VEC9 | SCO-spondin 547.50 | 5.66 | 2 | 2 |
| A8CG34 | Nuclear envelope pore membrane protein POM 121C 125.05 | 10.38 | 2 | 2 |
| O00116 | Alkyldihydroxyacetonephosphate synthase, peroxisomal 72.91 | 6.99 | 8 | 5 |
| O00400 | Acetyl-coenzyme A transporter 1 60.90 | 6.98 | 2 | 2 |
| O00410 | Importin-5 123.50 | 4.83 | 2 | 2 |
| O00469 | Procollagen-lysine,2-oxoglutarate 5-dioxygenase 2 82.03 | 6.16 | 13 | 6 |
| O00487 | 26S proteasome non-ATPase regulatory subunit 14 34.58 | 6.06 | 4 | 2 |
| O00767 | Acyl-CoA desaturase 41.52 | 9.07 | 3 | 2 |
| O14656 | Torsin-1A 35.78 | 6.16 | 3 | 2 |
| O14684 | Prostaglandin E synthase 17.10 | 9.59 | 6 | 3 |
| O15031 | Plexin-B2 203.25 | 5.85 | 5 | 2 |
| O15127 | Secretory carrier-associated membrane protein 2 36.65 | 5.72 | 3 | 2 |
| O15357 | Phosphatidylinositol-3,4,5-trisphosphate 5-phosphatase 2 138.60 | 6.1 | 2 | 2 |
| O15382 | Branched-chain-amino-acid aminotransferase, mitochondrial 41.26 | 8.21 | 4 | 3 |
| O15400 | Syntaxin-7 29.68 | 5.41 | 4 | 2 |
| O43151 | Probable methylcytosine dioxygenase TET3 179.35 | 7.01 | 2 | 2 |
| O43491 | Band 4.1-like protein 2 112.46 | 5.34 | 4 | 3 |
| O43837 | Isocitrate dehydrogenase [NAD] subunit beta, mitochondrial 38.79 | 7.82 | 8 | 4 |
| O60229 | Kalirin 340.17 | 5.72 | 2 | 2 |
| O60313 | Dynamin-like 120 kDa protein, mitochondrial 101.50 | 6.04 | 2 | 2 |
| O60488 | Long-chain-fatty-acid--CoA ligase 4 79.19 | 8.66 | 3 | 2 |
| O60664 | Perilipin-3 46.94 | 5.3 | 2 | 2 |
| O60716 | Catenin delta-1 108.17 | 5.86 | 5 | 2 |
| O60762 | Dolichol-phosphate mannosyltransferase 29.50 | 9.57 | 3 | 2 |
| O60831 | PRA1 family protein 2 19.26 | 9.41 | 11 | 2 |
| O60888 | Protein CutA 19.11 | 5.42 | 3 | 2 |
| O75439 | Mitochondrial-processing peptidase subunit beta 54.37 | 6.38 | 5 | 3 |
| O75445 | Usherin 575.60 | 6.4 | 2 | 2 |
| O75533 | Splicing factor 3B subunit 1 145.38 | 6.65 | 3 | 2 |
| O75643 | U5 small nuclear ribonucleoprotein 200 kDa helicase 244.51 | 5.73 | 4 | 4 |
| O75808 | Calpain-15 117.31 | 6.27 | 2 | 2 |
| O76024 | Wolframin 100.29 | 8.34 | 3 | 2 |
| O94874 | E3 UFM1-protein ligase 1 89.60 | 6.34 | 18 | 9 |
| O94905 | Erlin-2 37.84 | 5.47 | 2 | 2 |
| O95071 | E3 ubiquitin-protein ligase UBR5 309.35 | 5.59 | 3 | 3 |
| O95202 | LETM1 and EF-hand domain-containing protein 1, mitochondrial 83.35 | 6.3 | 10 | 6 |
| O95406 | Protein cornichon homolog 16.70 | 5.39 | 3 | 2 |
| O95571 | Protein ETHE1, mitochondrial 27.90 | 6.35 | 10 | 4 |
| O95777 | N-alpha-acetyltransferase 38, NatC auxiliary subunit 10.40 | 4.43 | 8 | 2 |
| O96005 | Cleft lip and palate transmembrane protein 1 76.10 | 5.88 | 4 | 3 |
| P01112 | GTPase HRas 21.30 | 5.16 | 2 | 2 |
| P04040 | Catalase 59.76 | 6.9 | 2 | 2 |
| P05023 | Sodium/potassium-transporting ATPase subunit alpha-1 112.90 | 5.33 | 26 | 12 |
| P05091 | Aldehyde dehydrogenase, mitochondrial 56.38 | 6.63 | 4 | 3 |
| P05783 | Keratin, type I cytoskeletal 18 48.06 | 5.34 | 20 | 6 |
| P05787 | Keratin, type II cytoskeletal 8 53.70 | 5.52 | 19 | 9 |
| P05997 | Collagen alpha-2(V) chain 144.91 | 6.07 | 2 | 2 |
| P07099 | Epoxide hydrolase 1 52.95 | 6.77 | 4 | 4 |
| P07384 | Calpain-1 catalytic subunit 81.89 | 5.49 | 2 | 2 |
| P07910 | Heterogeneous nuclear ribonucleoproteins C1/C2 33.67 | 4.95 | 3 | 3 |
| P07942 | Laminin subunit beta-1 198.04 | 4.83 | 6 | 3 |
| P07996 | Thrombospondin-1 129.38 | 4.71 | 3 | 3 |
| P08559 | Pyruvate dehydrogenase E1 component subunit alpha, somatic form, mitoc43.30 | 8.35 | 6 | 5 |
| P08621 | U1 small nuclear ribonucleoprotein 70 kDa 51.56 | 9.94 | 6 | 2 |
| P08670 | Vimentin 53.65 | 5.05 | 6 | 3 |
| P08727 | Keratin, type I cytoskeletal 19 44.10 | 5.05 | 26 | 14 |
| P08729 | Keratin, type II cytoskeletal 7 51.39 | 5.39 | 12 | 7 |
| P08754 | Guanine nucleotide-binding protein G(k) subunit alpha; G(i) alpha-3 40.53 | 5.5 | 14 | 6 |
| P09110 | 3-ketoacyl-CoA thiolase, peroxisomal 44.29 | 8.76 | 2 | 2 |
| P10155 | 60 kDa SS-A/Ro ribonucleoprotein 60.67 | 8.27 | 3 | 2 |
| P10515 | Dihydrolipoyllysine-residue acetyltransferase component of pyruvate dehyd 69.00 | 7.96 | 6 | 3 |
| P10586 | Receptor-type tyrosine-protein phosphatase F 212.88 | 5.92 | 2 | 2 |
| P10599 | Thioredoxin 11.73 | 4.82 | 3 | 2 |
| P10620 | Microsomal glutathione S-transferase 1 17.60 | 9.41 | 27 | 7 |
| P11166 | Solute carrier family 2, facilitated glucose transporter member 1 54.08 | 8.93 | 5 | 3 |
| P11177 | Pyruvate dehydrogenase E1 component subunit beta, mitochondrial 39.23 | 6.2 | 14 | 6 |
| P11310 | Medium-chain specific acyl-CoA dehydrogenase, mitochondrial 46.59 | 8.61 | 5 | 2 |
| P12111 | Collagen alpha-3(VI) chain 343.67 | 6.26 | 2 | 2 |
| P12270 | Nucleoprotein TPR 267.29 | 4.97 | 4 | 3 |
| P13473 | Lysosome-associated membrane glycoprotein 2 44.96 | 5.35 | 6 | 3 |
| P13646 | Keratin, type I cytoskeletal 13 49.59 | 4.91 | 2 | 2 |
| P13861 | cAMP-dependent protein kinase type II-alpha regulatory subunit 45.52 | 4.96 | 2 | 2 |
| P14923 | Junction plakoglobin 81.74 | 5.75 | 6 | 4 |
| P15144 | Aminopeptidase N 109.54 | 5.31 | 3 | 2 |
| P15924 | Desmoplakin 331.77 | 6.44 | 5 | 4 |
| P18462 | HLA class I histocompatibility antigen, A-25 alpha chain 41.22 | 6.09 | 2 | 2 |
| P20290 | Transcription factor BTF3 22.17 | 9.41 | 3 | 2 |
| P20339 | Ras-related protein Rab-5A 23.66 | 8.32 | 3 | 2 |
| P20674 | Cytochrome c oxidase subunit 5A, mitochondrial 16.76 | 6.3 | 6 | 3 |
| P20700 | Lamin-B1 66.41 | 5.11 | 4 | 2 |
| P20908 | Collagen alpha-1(V) chain 183.56 | 4.94 | 3 | 3 |
| P21796 | Voltage-dependent anion-selective channel protein 1 30.77 | 8.62 | 15 | 7 |
| P22033 | Methylmalonyl-CoA mutase, mitochondrial 83.13 | 6.48 | 3 | 2 |
| P22307 | Non-specific lipid-transfer protein 58.99 | 6.44 | 9 | 5 |
| P22392 | Nucleoside diphosphate kinase B 17.30 | 8.52 | 2 | 2 |
| P22626 | Heterogeneous nuclear ribonucleoproteins A2/B1 37.43 | 8.97 | 9 | 6 |
| P23470 | Receptor-type tyrosine-protein phosphatase gamma 162.00 | 5.99 | 2 | 2 |
| P23634 | Plasma membrane calcium-transporting ATPase 4 137.92 | 6.19 | 7 | 2 |
| P25205 | DNA replication licensing factor MCM3 90.98 | 5.53 | 3 | 2 |
| P26006 | Integrin alpha-3 116.61 | 6.32 | 3 | 2 |
| P27144 | Adenylate kinase isoenzyme 4, mitochondrial 25.27 | 8.47 | 3 | 2 |
| P27449 | V-type proton ATPase 16 kDa proteolipid subunit 15.74 | 7.98 | 4 | 2 |
| P27695 | DNA-(apurinic or apyrimidinic site) lyase 35.55 | 8.33 | 2 | 2 |
| P28838 | Cytosol aminopeptidase 56.17 | 8.03 | 2 | 2 |
| P29317 | Ephrin type-A receptor 2 108.27 | 5.86 | 2 | 2 |
| P29966 | Myristoylated alanine-rich C-kinase substrate 31.55 | 4.46 | 3 | 2 |
| P30042 | ES1 protein homolog, mitochondrial 28.17 | 8.5 | 4 | 2 |
| P30049 | ATP synthase subunit delta, mitochondrial 17.50 | 5.34 | 10 | 3 |
| P30462 | HLA class I histocompatibility antigen, B-14 alpha chain 40.36 | 5.56 | 3 | 3 |

| P30837 | Aldehyde dehydrogenase X, mitochondrial 57.21 | 6.36 | 5 | 3 |
| --- | --- | --- | --- | --- |
| P31930 | Cytochrome b-c1 complex subunit 1, mitochondrial 52.65 | 5.94 | 11 | 4 |
| P32004 | Neural cell adhesion molecule L1 140.00 | 5.84 | 18 | 11 |
| P32119 | Peroxiredoxin-2 21.89 | 5.66 | 5 | 2 |
| P33991 | DNA replication licensing factor MCM4 96.56 | 6.28 | 2 | 2 |
| P33992 | DNA replication licensing factor MCM5 82.29 | 8.64 | 3 | 2 |
| P33993 | DNA replication licensing factor MCM7 81.30 | 6.08 | 12 | 6 |
| P34932 | Heat shock 70 kDa protein 4 94.33 | 5.1 | 5 | 3 |
| P35222 | Catenin beta-1 85.50 | 5.53 | 13 | 8 |
| P35637 | RNA-binding protein FUS 53.43 | 9.4 | 2 | 2 |
| P37268 | Squalene synthase 48.12 | 6.1 | 9 | 4 |
| P38919 | Eukaryotic initiation factor 4A-III 46.87 | 6.3 | 2 | 2 |
| P42694 | Probable helicase with zinc finger domain 219.00 | 7.04 | 2 | 2 |
| P43307 | Translocon-associated protein subunit alpha 32.24 | 4.37 | 10 | 3 |
| P43897 | Elongation factor Ts, mitochondrial 35.40 | 8.62 | 4 | 4 |
| P45880 | Voltage-dependent anion-selective channel protein 2 31.57 | 7.5 | 3 | 3 |
| P46459 | Vesicle-fusing ATPase 82.59 | 6.25 | 7 | 6 |
| P46821 | Microtubule-associated protein 1B 270.63 | 4.73 | 3 | 3 |
| P47897 | Glutaminyl-tRNA synthetase 87.80 | 6.71 | 3 | 2 |
| P48449 | Lanosterol synthase 83.33 | 6.16 | 11 | 8 |
| P48735 | Isocitrate dehydrogenase [NADP], mitochondrial 50.91 | 8.88 | 18 | 8 |
| P49419 | Alpha-aminoadipic semialdehyde dehydrogenase 55.49 | 8.21 | 12 | 9 |
| P49748 | Very long-chain specific acyl-CoA dehydrogenase, mitochondrial 70.39 | 8.92 | 13 | 6 |
| P51114 | Fragile X mental retardation syndrome-related protein 1 69.72 | 5.84 | 2 | 2 |
| P51659 | Peroxisomal multifunctional enzyme type 2 79.69 | 8.96 | 3 | 2 |
| P51665 | 26S proteasome non-ATPase regulatory subunit 7 37.03 | 6.29 | 3 | 2 |
| P51991 | Heterogeneous nuclear ribonucleoprotein A3 39.59 | 9.1 | 3 | 3 |
| P52292 | Importin subunit alpha-2 57.86 | 5.25 | 8 | 4 |
| P52815 | 39S ribosomal protein L12, mitochondrial 21.35 | 9.05 | 3 | 2 |
| P53985 | Monocarboxylate transporter 1 53.94 | 8.91 | 3 | 2 |
| P54652 | Heat shock-related 70 kDa protein 2 70.02 | 5.55 | 2 | 2 |
| P54886 | Delta-1-pyrroline-5-carboxylate synthase 87.30 | 6.66 | 40 | 17 |
| P56696 | Potassium voltage-gated channel subfamily KQT member 4 77.10 | 9.6 | 2 | 2 |
| P57105 | Synaptojanin-2-binding protein 15.93 | 5.86 | 4 | 2 |
| P60033 | CD81 antigen 25.81 | 5.09 | 4 | 2 |
| P61006 | Ras-related protein Rab-8A 23.67 | 9.15 | 2 | 2 |
| P61020 | Ras-related protein Rab-5B 23.71 | 8.29 | 6 | 2 |
| P61513 | 60S ribosomal protein L37a 10.28 | 10.44 | 3 | 2 |
| P61586 | Transforming protein RhoA 21.77 | 5.83 | 6 | 4 |
| P62070 | Ras-related protein R-Ras2 23.40 | 5.74 | 3 | 2 |
| P62158 | Calmodulin 16.84 | 4.09 | 3 | 2 |
| P62266 | 40S ribosomal protein S23 15.81 | 10.5 | 9 | 3 |
| P62330 | ADP-ribosylation factor 6 20.08 | 9.04 | 3 | 2 |
| P62341 | Selenoprotein T 22.32 | 8.79 | 2 | 2 |
| P67812 | Signal peptidase complex catalytic subunit SEC11A 20.62 | 9.48 | 3 | 2 |
| P68371 | Tubulin beta-2C chain 49.83 | 4.79 | 6 | 2 |
| P82650 | 28S ribosomal protein S22, mitochondrial 41.28 | 7.7 | 3 | 2 |
| Q00325 | Phosphate carrier protein, mitochondrial 40.09 | 9.45 | 6 | 4 |
| Q00765 | Receptor expression-enhancing protein 5 21.49 | 8.25 | 2 | 2 |
| Q01081 | Splicing factor U2AF 35 kDa subunit 27.87 | 9.09 | 2 | 2 |
| Q01628 | Interferon-induced transmembrane protein 3 14.63 | 6.49 | 5 | 2 |
| Q02809 | Procollagen-lysine,2-oxoglutarate 5-dioxygenase 1 83.55 | 6.46 | 3 | 2 |
| Q02818 | Nucleobindin-1 53.88 | 5.15 | 6 | 2 |
| Q02978 | Mitochondrial 2-oxoglutarate/malate carrier protein 34.06 | 9.92 | 9 | 5 |
| Q06136 | 3-ketodihydrosphingosine reductase 36.19 | 6.75 | 3 | 2 |
| Q08211 | ATP-dependent RNA helicase A 140.96 | 6.41 | 3 | 2 |
| Q08380 | Galectin-3-binding protein 65.33 | 5.12 | 9 | 4 |
| Q08945 | FACT complex subunit SSRP1 81.07 | 6.44 | 3 | 2 |
| Q12907 | Vesicular integral-membrane protein VIP36 40.23 | 6.46 | 6 | 2 |
| Q12931 | Heat shock protein 75 kDa, mitochondrial 80.11 | 8.3 | 14 | 9 |
| Q13045 | Protein flightless-1 homolog 144.75 | 5.75 | 4 | 2 |
| Q13151 | Heterogeneous nuclear ribonucleoprotein A0 30.84 | 9.34 | 2 | 2 |
| Q13200 | 26S proteasome non-ATPase regulatory subunit 2 100.20 | 5.08 | 3 | 2 |
| Q13405 | 39S ribosomal protein L49, mitochondrial 19.20 | 9.47 | 2 | 2 |
| Q13724 | Mannosyl-oligosaccharide glucosidase 91.92 | 8.97 | 5 | 3 |
| Q13740 | CD166 antigen 65.10 | 5.92 | 11 | 6 |
| Q14165 | Malectin 32.23 | 5.27 | 6 | 3 |
| Q14240 | Eukaryotic initiation factor 4A-II 46.40 | 5.33 | 2 | 2 |
| Q14573 | Inositol 1,4,5-trisphosphate receptor type 3 304.11 | 6.05 | 4 | 3 |
| Q14839 | Chromodomain-helicase-DNA-binding protein 4 218.00 | 5.62 | 2 | 2 |
| Q15006 | Tetratricopeptide repeat protein 35 34.83 | 6.15 | 3 | 2 |
| Q15041 | ADP-ribosylation factor-like protein 6-interacting protein 1 23.36 | 9.38 | 4 | 2 |
| Q15056 | Eukaryotic translation initiation factor 4H 27.39 | 6.66 | 4 | 2 |
| Q15125 | 3-beta-hydroxysteroid-Delta(8),Delta(7)-isomerase 26.35 | 7.76 | 6 | 2 |
| Q15155 | Nodal modulator 1 134.32 | 5.54 | 6 | 5 |
| Q15165 | Serum paraoxonase/arylesterase 2 39.40 | 5.33 | 4 | 2 |
| Q15363 | Transmembrane emp24 domain-containing protein 2 22.76 | 5.08 | 18 | 3 |
| Q15596 | Nuclear receptor coactivator 2 159.16 | 6.19 | 3 | 3 |
| Q16585 | Beta-sarcoglycan 34.78 | 8.86 | 4 | 2 |
| Q16718 | NADH dehydrogenase [ubiquinone] 1 alpha subcomplex subunit 5 13.46 | 5.75 | 4 | 2 |
| Q17RC7 | SEC6-like protein C14orf73 79.89 | 5.92 | 2 | 2 |
| Q3ZAQ7 | Vacuolar ATPase assembly integral membrane protein VMA21 11.35 | 6.55 | 4 | 2 |
| Q3ZCQ8 | Mitochondrial import inner membrane translocase subunit TIM50 39.65 | 8.55 | 7 | 2 |
| Q56VL3 | OCIA domain-containing protein 2 16.95 | 9.24 | 3 | 2 |
| Q5JRX3 | Presequence protease, mitochondrial 117.41 | 6.45 | 3 | 2 |
| Q5JTZ9 | Probable alanyl-tRNA synthetase, mitochondrial 107.34 | 5.87 | 4 | 2 |
| Q5JWF2 | Guanine nucleotide-binding protein G(s) subunit alpha isoforms XLas 111.02 | 4.91 | 4 | 3 |
| Q5SRD1 | Putative mitochondrial import inner membrane translocase subunit Tim23B 28.05 | 9.47 | 4 | 2 |
| Q5T1B0 | Uncharacterized protein C1orf125 118.03 | 5.49 | 2 | 2 |
| Q5T4S7 | E3 ubiquitin-protein ligase UBR4.. 573.84 | 5.7 | 2 | 2 |
| Q5ZPR3 | CD276 antigen 57.24 | 4.77 | 4 | 2 |
| Q6P2Q9 | Pre-mRNA-processing-splicing factor 8 273.60 | 8.95 | 3 | 2 |
| Q6UB35 | Monofunctional C1-tetrahydrofolate synthase, mitochondrial 105.79 | 8.32 | 5 | 3 |
| Q6YHK3 | CD109 antigen 161.69 | 5.59 | 4 | 2 |
| Q6YN16 | Hydroxysteroid dehydrogenase-like protein 2 45.39 | 8.07 | 4 | 2 |
| Q709C8 | Vacuolar protein sorting-associated protein 13C 422.39 | 6.38 | 2 | 2 |
| Q7Z6L1 | Tectonin beta-propeller repeat-containing protein 1 129.70 | 5.83 | 2 | 2 |
| Q86Y82 | Syntaxin-12 31.64 | 5.45 | 6 | 4 |
| Q8N2C7 | Protein unc-80 homolog 363.39 | 6.4 | 2 | 2 |
| Q8N3C0 | Activating signal cointegrator 1 complex subunit 3 251.46 | 6.64 | 2 | 2 |
| Q8N766 | Uncharacterized protein KIAA0090 111.76 | 7.38 | 6 | 4 |
| Q8NHH9 | Atlastin-2 66.23 | 5.3 | 3 | 2 |
| Q8NHW5 | 60S acidic ribosomal protein P0-like 34.36 | 5.39 | 5 | 4 |

| Q8TCT9 | Minor histocompatibility antigen H13 | 41.49 | 6 | 3 | 2 |
| --- | --- | --- | --- | --- | --- |
| Q8WVM8 | Sec1 family domain-containing protein 1 | 72.38 | 5.89 | 8 | 4 |
| Q92542 | Nicastrin | 78.41 | 5.67 | 4 | 2 |
| Q92896 | Golgi apparatus protein 1 | 134.55 | 6.52 | 2 | 2 |
| Q969U6 | F-box/WD repeat-containing protein 5 | 63.92 | 5.89 | 2 | 2 |
| Q96CS3 | FAS-associated factor 2 | 52.62 | 5.45 | 2 | 2 |
| Q96EA4 | Protein Spindly | 70.17 | 5.41 | 2 | 2 |
| Q96I99 | Succinyl-CoA ligase [GDP-forming] subunit beta, mitochondrial | 46.51 | 6.15 | 9 | 5 |
| Q96PK2 | Microtubule-actin cross-linking factor 1, isoform 4 | 838.31 | 5.28 | 4 | 4 |
| Q96Q15 | Serine/threonine-protein kinase SMG1 | 410.50 | 6.03 | 3 | 2 |
| Q96RQ3 | Methylcrotonoyl-CoA carboxylase subunit alpha, mitochondrial | 80.47 | 7.66 | 3 | 2 |
| Q96RT1 | Protein LAP2; Erbb2-interacting protein | 158.30 | 5.32 | 4 | 3 |
| Q99501 | GAS2-like protein 1 | 72.72 | 10.1 | 2 | 2 |
| Q99623 | Prohibitin-2; B-cell receptor-associated protein BAP37 | 33.30 | 9.83 | 26 | 10 |
| Q99798 | Aconitate hydratase, mitochondrial | 85.43 | 7.36 | 31 | 13 |
| Q99805 | Transmembrane 9 superfamily member 2 | 75.78 | 7.22 | 2 | 2 |
| Q9BSV6 | tRNA-splicing endonuclease subunit Sen34 | 33.65 | 8.53 | 2 | 2 |
| Q9BTC0 | Death-inducer obliterator 1 | 243.87 | 8.09 | 3 | 2 |
| Q9BTT6 | Leucine-rich repeat-containing protein 1 | 59.24 | 4.94 | 3 | 2 |
| Q9BWM7 | Sideroflexin-3 | 35.98 | 9.25 | 3 | 2 |
| Q9BYT8 | Neurolysin, mitochondrial | 80.65 | 6.21 | 3 | 2 |
| Q9H3Z4 | DnaJ homolog subfamily C member 5 | 22.15 | 4.93 | 5 | 3 |
| Q9H6S3 | Epidermal growth factor receptor kinase substrate 8-like protein 2 | 80.62 | 6.39 | 2 | 2 |
| Q9H7P9 | Pleckstrin homology domain-containing family G member 2 | 147.97 | 5.57 | 2 | 2 |
| Q9H7Z7 | Prostaglandin E synthase 2 | 41.94 | 9.22 | 4 | 3 |
| Q9H845 | Acyl-CoA dehydrogenase family member 9, mitochondrial | 68.76 | 8.15 | 4 | 2 |
| Q9HAV7 | GrpE protein homolog 1, mitochondrial | 24.28 | 8.24 | 2 | 2 |
| Q9HCC0 | Methylcrotonoyl-CoA carboxylase beta chain, mitochondrial | 61.33 | 7.58 | 7 | 4 |
| Q9HCM3 | UPF0606 protein KIAA1549 | 210.75 | 5.75 | 3 | 3 |
| Q9NR31 | GTP-binding protein SAR1a; COPII-associated small GTPase | 22.37 | 6.21 | 5 | 2 |
| Q9NS69 | Mitochondrial import receptor subunit TOM22 homolog | 15.52 | 4.27 | 4 | 2 |
| Q9NSE4 | Isoleucyl-tRNA synthetase, mitochondrial | 113.79 | 6.78 | 16 | 9 |
| Q9NTG7 | NAD-dependent deacetylase sirtuin-3, mitochondrial | 43.57 | 8.98 | 3 | 2 |
| Q9NTK5 | Obg-like ATPase 1 | 44.74 | 7.64 | 2 | 2 |
| Q9NU22 | Midasin | 632.82 | 5.46 | 2 | 2 |
| Q9NX62 | Inositol monophosphatase 3 | 38.58 | 6.38 | 3 | 3 |
| Q9P000 | COMM domain-containing protein 9 | 21.82 | 5.6 | 4 | 2 |
| Q9P0L0 | Vesicle-associated membrane protein-associated protein A | 27.89 | 8.8 | 7 | 3 |
| Q9P258 | Protein RCC2 | 56.08 | 9.02 | 2 | 2 |
| Q9P2B2 | Prostaglandin F2 receptor negative regulator | 98.56 | 6.16 | 4 | 2 |
| Q9P2E9 | Ribosome-binding protein 1 | 152.47 | 8.69 | 8 | 4 |
| Q9P2R7 | Succinyl-CoA ligase [ADP-forming] subunit beta, mitochondrial | 50.32 | 7.05 | 3 | 3 |
| Q9UBM7 | 7-dehydrocholesterol reductase | 54.49 | 8.95 | 4 | 2 |
| Q9UBQ7 | Glyoxylate reductase/hydroxypyruvate reductase | 35.67 | 7.01 | 3 | 2 |
| Q9UGP8 | Translocation protein SEC63 homolog | 87.99 | 5.21 | 12 | 6 |
| Q9UH99 | SUN domain-containing protein 2 | 80.31 | 6.27 | 3 | 2 |
| Q9UHA4 | Mitogen-activated protein kinase scaffold protein 1 | 13.62 | 6.72 | 5 | 2 |
| Q9UHG3 | Prenylcysteine oxidase 1 | 56.64 | 5.8 | 3 | 3 |
| Q9UIQ6 | Leucyl-cystinyl aminopeptidase | 117.35 | 5.5 | 3 | 2 |
| Q9UJS0 | Calcium-binding mitochondrial carrier protein Aralar2 | 74.18 | 8.79 | 8 | 4 |
| Q9UJZ1 | Stomatin-like protein 2 | 38.53 | 6.87 | 38 | 6 |
| Q9UMD9 | Collagen alpha-1(XVII) chain | 150.42 | 8.89 | 4 | 4 |
| Q9UNM6 | 26S proteasome non-ATPase regulatory subunit 13 | 42.95 | 5.53 | 2 | 2 |
| Q9UPN3 | Microtubule-actin cross-linking factor 1, isoforms 1/2/3/5 | 838.31 | 5.28 | 3 | 2 |
| Q9UPT8 | Zinc finger CCCH domain-containing protein 4 | 140.26 | 5.87 | 2 | 2 |
| Q9UPX8 | SH3 and multiple ankyrin repeat domains protein 2 | 158.82 | 6.48 | 2 | 2 |
| Q9Y2Q3 | Glutathione S-transferase kappa 1 | 25.50 | 8.51 | 6 | 2 |
| Q9Y2W1 | Thyroid hormone receptor-associated protein 3 | 108.67 | 10.16 | 3 | 2 |
| Q9Y305 | Acyl-coenzyme A thioesterase 9, mitochondrial | 49.90 | 8.81 | 3 | 2 |
| Q9Y3E5 | Peptidyl-tRNA hydrolase 2, mitochondrial | 19.19 | 8.95 | 3 | 2 |
| Q9Y4A5 | Transformation/transcription domain-associated protein | 437.60 | 8.49 | 2 | 2 |
| Q9Y5M8 | Signal recognition particle receptor subunit beta | 29.70 | 9.17 | 3 | 3 |
| Q9Y624 | Junctional adhesion molecule A | 32.58 | 8.09 | 3 | 2 |
| Q9Y6C9 | Mitochondrial carrier homolog 2 | 33.33 | 8.25 | 46 | 8 |
