# Supplementary material for: Syntenin-1 is a promoter and prognostic marker of head and neck squamous cell carcinoma invasion and metastasis
Source: Oncotarget. 2016 Nov 2;7(50):82634–47. doi: 10.18632/oncotarget.13020 (PMC5347720; doi:10.18632/oncotarget.13020)
Supplement: Supplementary file 4 [file oncotarget-07-82634-s004.docx]

**Supplemental Table 3: Membrane and membrane-associated proteins identified both in UM1 and UM2 cells.**

Accession Protein name Molecular Weight (KDa) PI UM1(Nubmers of total peptides) UM1 (Nubmers of unique peptides) UM2(Nubmers of total peptides) UM2(Nubmers of unique peptides)

| Q9Y678 | Coatomer subunit gamma | 97.72 | 5.32 | 5 | 3 | 6 | 5 |
| --- | --- | --- | --- | --- | --- | --- | --- |
| Q9Y4L1 | Hypoxia up-regulated protein 1 | 107.66 | 5.07 | 35 | 17 | 44 | 18 |
| Q9Y3D6 | Mitochondrial fission 1 protein | 16.94 | 8.84 | 3 | 2 | 16 | 2 |
| Q9Y3B3 | Transmembrane emp24 domain-containing protein 7 | 21.7 | 5.41 | 3 | 3 | 7 | 3 |
| Q9Y3A6 | Transmembrane emp24 domain-containing protein 5 | 23.25 | 4.7 | 2 | 2 | 8 | 2 |
| Q9Y394 | Dehydrogenase/reductase SDR family member 7 | 34.94 | 8.46 | 24 | 6 | 11 | 5 |
| Q9Y2X9 | Zinc finger protein 281 | 96.91 | 8.71 | 2 | 2 | 2 | 2 |
| Q9Y224 | UPF0568 protein C14orf166 | 28.07 | 6.19 | 6 | 3 | 2 | 2 |
| Q9UL46 | Proteasome activator complex subunit 2 | 27.27 | 5.54 | 9 | 3 | 8 | 3 |
| Q9UIJ7 | GTP:AMP phosphotransferase mitochondrial | 25.43 | 9.16 | 11 | 5 | 8 | 5 |
| Q9UL25 | Ras-related protein Rab-21 | 23.99 | 8.16 | 4 | 2 | 4 | 2 |
| Q9UBI6 | Guanine nucleotide-binding protein G(I)/G(S)/G(O) subunit gamma-12 | 7.54 | 9.14 | 3 | 2 | 4 | 2 |
| Q9P0S9 | Transmembrane protein 14C | 11.56 | 9.87 | 4 | 2 | 8 | 3 |
| Q9P035 | Protein tyrosine phosphatase-like protein PTPLAD1 | 43.16 | 9.04 | 2 | 2 | 5 | 2 |
| Q9NZM1 | Myoferlin | 234.71 | 5.84 | 13 | 9 | 82 | 39 |
| Q9NYU2 | UDP-glucose:glycoprotein glucosyltransferase 1 | 173.03 | 5.38 | 9 | 4 | 15 | 7 |
| Q9NQC3 | Reticulon-4 | 129.93 | 4.42 | 183 | 8 | 29 | 8 |
| Q9HDC9 | Adipocyte plasma membrane-associated protein | 46.35 | 5.82 | 6 | 5 | 6 | 5 |
| Q9HCY8 | Protein S100-A14 | 11.66 | 5.16 | 4 | 2 | 3 | 2 |
| Q9H9P8 | L-2-hydroxyglutarate dehydrogenase, mitochondrial | 45.29 | 7.14 | 3 | 2 | 3 | 2 |
| Q9H9B4 | Sideroflexin-1 | 35.49 | 9.22 | 4 | 2 | 4 | 2 |
| Q9H488 | GDP-fucose protein O-fucosyltransferase 1 | 41.28 | 8.65 | 6 | 2 | 4 | 3 |
| Q9H3N1 | Thioredoxin-related transmembrane protein 1 | 29.15 | 4.86 | 6 | 3 | 9 | 5 |
| Q9H2U2 | Inorganic pyrophosphatase 2, mitochondrial | 34.71 | 5.97 | 8 | 4 | 8 | 4 |
| Q9H0U4 | Ras-related protein Rab-1B | 22.17 | 5.55 | 20 | 7 | 112 | 11 |
| Q9BX68 | Histidine triad nucleotide-binding protein 2, mitochondria | 15.51 | 7.13 | 5 | 2 | 7 | 2 |
| Q9BVK6 | Transmembrane emp24 domain-containing protein 9 | 23.32 | 6.2 | 24 | 3 | 10 | 5 |
| Q9BTV4 | Transmembrane protein 43 | 44.74 | 8.01 | 3 | 2 | 3 | 2 |
| Q9BSJ8 | Extended synaptotagmin-1 | 122.86 | 5.57 | 19 | 10 | 48 | 20 |
| Q9BS26 | Endoplasmic reticulum resident protein 44 | 43.72 | 5.04 | 6 | 2 | 8 | 3 |
| Q99832 | T-complex protein 1 subunit eta | 59.37 | 7.55 | 15 | 7 | 10 | 4 |
| Q99714 | 3-hydroxyacyl-CoA dehydrogenase type-2 | 26.79 | 7.87 | 23 | 7 | 21 | 5 |
| Q99653 | Calcium-binding protein p22 | 22.33 | 4.98 | 2 | 2 | 2 | 2 |
| Q99447 | Ethanolamine-phosphate cytidylyltransferase | 43.84 | 6.44 | 8 | 2 | 3 | 2 |
| Q96RP9 | Elongation factor G, mitochondrial | 7955 | 5.9 | 3 | 3 | 16 | 10 |
| Q96QK1 | Vacuolar protein sorting-associated protein 35 | 91.71 | 5.32 | 6 | 3 | 6 | 3 |
| Q96IX5 | Up-regulated during skeletal muscle growth protein 5 | 6.33 | 9.78 | 4 | 2 | 3 | 2 |
| Q96HE7 | ERO1-like protein alpha | 51.99 | 5.37 | 28 | 10 | 14 | 8 |
| Q96FW1 | Ubiquitin thioesterase OTUB1 | 31.15 | 4.85 | 4 | 2 | 4 | 2 |
| Q96BM9 | ADP-ribosylation factor-like protein 8A | 21.42 | 7.63 | 10 | 3 | 8 | 4 |
| Q96AG4 | Leucine-rich repeat-containing protein 59 | 34.93 | 9.61 | 6 | 4 | 16 | 7 |
| Q96AB3 | Isochorismatase domain-containing protein 2, mitochondrial | 22.34 | 7.67 | 5 | 3 | 5 | 3 |
| Q969H8 | UPF0556 protein C19orf10 | 15.83 | 6.22 | 6 | 2 | 6 | 3 |
| Q969G5 | Protein kinase C delta-binding protein | 27.7 | 6.05 | 2 | 2 | 2 | 2 |
| Q92616 | Translational activator GCN1 | 292.6 | 7.32 | 6 | 4 | 15 | 8 |
| Q92520 | Protein FAM3C | 22.16 | 7.75 | 7 | 2 | 2 | 2 |
| Q92499 | ATP-dependent RNA helicase DDX1 | 82.43 | 6.8 | 5 | 2 | 5 | 2 |
| Q8WXH0 | Nesprin-2 | 796.44 | 5.26 | 3 | 2 | 6 | 6 |
| Q8TC12 | Retinol dehydrogenase 11 | 35.39 | 9.05 | 2 | 2 | 4 | 2 |
| Q8NF37 | Lysophosphatidylcholine acyltransferase 1 | 59.15 | 5.77 | 4 | 3 | 5 | 2 |
| Q8NBX0 | Probable saccharopine dehydrogenase | 47.02 | 9.24 | 8 | 2 | 20 | 8 |
| Q8NBS9 | Thioredoxin domain-containing protein 5 | 44.46 | 5.37 | 9 | 5 | 9 | 5 |
| Q86VP6 | Cullin-associated NEDD8-dissociated protein 1 | 136.24 | 5.52 | 3 | 2 | 2 | 2 |
| Q86UU0 | B-cell CLL/lymphoma 9-like protein | 157.13 | 8.79 | 2 | 2 | 3 | 3 |
| Q86UP2 | Kinectin | 156.28 | 5.52 | 6 | 4 | 6 | 4 |
| Q86UE4 | Protein LYRIC | 63.84 | 9.33 | 5 | 3 | 4 | 2 |
| Q7L1Q6 | Basic leucine zipper and W2 domain-containing protein 1 | 48.04 | 5.74 | 7 | 3 | 3 | 2 |
| Q7KZF4 | Staphylococcal nuclease domain-containing protein 1 | 101.87 | 6.77 | 15 | 9 | 15 | 9 |
| Q71U36 | Tubulin alpha-1A chain | 50.14 | 4.94 | 3 | 3 | 4 | 3 |
| Q6PIU2 | Neutral cholesterol ester hydrolase 1 | 45.81 | 6.76 | 5 | 3 | 17 | 8 |
| Q6NZI2 | Polymerase I and transcript release factor | 43.48 | 5.5 | 9 | 3 | 20 | 7 |
| Q6NUK1 | Calcium-binding mitochondrial carrier protein SCaMC-1 | 53.35 | 6 | 3 | 2 | 3 | 2 |
| Q6DD88 | Atlastin-3 | 60.54 | 5.43 | 6 | 5 | 19 | 10 |
| S7N6L8 | Putative small nuclear ribonucleoprotein polypeptide E-like protein 1 | 8 | 6.78 | 5 | 2 | 5 | 3 |
| Q562R1 | Beta-actin-like protein 2 | 42 | 5.39 | 19 | 2 | 4 | 2 |
| Q53GQ0 | Estradiol 17-beta-dehydrogenase 12 | 34.32 | 9.34 | 12 | 7 | 19 | 8 |
| Q27J81 | Inverted formin-2 | 135.49 | 5.26 | 3 | 2 | 10 | 6 |
| Q16850 | Lanosterol 14-alpha demethylase | 56.81 | 8.72 | 4 | 3 | 5 | 2 |
| Q16836 | Hydroxyacyl-coenzyme A dehydrogenase, mitochondrial | 32.84 | 8.38 | 2 | 2 | 16 | 6 |
| Q16822 | Phosphoenolpyruvate carboxykinase [GTP], mitochondrial | 67.1 | 6.58 | 4 | 2 | 4 | 2 |
| Q16531 | DNA damage-binding protein 1 | 126.84 | 5.14 | 3 | 2 | 3 | 3 |
| Q15907 | Ras-related protein Rab-11B | 24 | 5.65 | 9 | 5 | 16 | 8 |
| Q15836 | Vesicle-associated membrane protein 3 | 11.18 | 8.89 | 11 | 4 | 11 | 4 |
| Q15758 | Neutral amino acid transporter B(0) | 56.6 | 5.34 | 6 | 4 | 9 | 4 |
| Q15738 | Sterol-4-alpha-carboxylate 3-dehydrogenase, decarboxylating | 41.9 | 8.16 | 8 | 4 | 8 | 4 |
| Q15366 | Poly(rC)-binding protein 2 | 38.58 | 6.33 | 7 | 6 | 3 | 2 |
| Q15149 | Plectin | 531.8 | 5.74 | 353 | 139 | 231 | 116 |
| Q15084 | Protein disulfide-isomerase A6 | 46.17 | 4.95 | 33 | 11 | 102 | 12 |
| Q14980 | Nuclear mitotic apparatus protein 1 | 238.26 | 5.63 | 7 | 6 | 20 | 11 |
| Q14974 | Importin subunit beta-1 | 97.17 | 4.68 | 10 | 5 | 10 | 5 |
| Q14697 | Neutral alpha-glucosidase AB | 103.95 | 5.58 | 14 | 7 | 56 | 20 |
| Q14257 | Reticulocalbin-2 | 34.78 | 4.22 | 6 | 5 | 3 | 2 |
| Q14204 | Cytoplasmic dynein 1 heavy chain 1 | 532.28 | 6.01 | 31 | 21 | 28 | 16 |
| Q13838 | Spliceosome RNA helicase BAT1 | 48.86 | 5.44 | 2 | 2 | 3 | 2 |
| Q13813 | Spectrin alpha chain, brain | 284.54 | 5.22 | 22 | 11 | 22 | 11 |
| Q13753 | Laminin subunit gamma-2 | 128.74 | 5.79 | 9 | 6 | 6 | 4 |
| Q13751 | Laminin subunit beta-3 | 127.64 | 7.01 | 48 | 5 | 48 | 5 |
| Q13637 | Ras-related protein Rab-32 | 25 | 6.1 | 4 | 2 | 3 | 2 |
| Q13263 | Transcription intermediary factor 1-beta | 88.42 | 5.52 | 4 | 4 | 15 | 9 |
| Q13162 | Peroxiredoxin-4 | 26.57 | 5.54 | 11 | 3 | 31 | 10 |
| Q13011 | Delta(3,5)-Delta(2,4)-dienoyl-CoA isomerase, mitochondrial | 32.2 | 5.99 | 3 | 2 | 16 | 7 |
| Q10713 | Mitochondrial-processing peptidase subunit alpha | 54.67 | 5.88 | 3 | 3 | 5 | 3 |
| Q07065 | Cytoskeleton-associated protein 4 | 66.02 | 5.63 | 16 | 8 | 15 | 6 |
| Q07021 | Complement component 1 Q subcomponent-binding protein, mitochondrial | 23.78 | 4.32 | 23 | 9 | 23 | 9 |
| Q07020 | 60S ribosomal protein L18 | 21.5 | 11.73 | 10 | 3 | 3 | 2 |
| Q06323 | Proteasome activator complex subunit 1 | 28.72 | 5.78 | 6 | 3 | 6 | 3 |
| Q04941 | Proteolipid protein 2 | 16.69 | 6.8 | 4 | 2 | 6 | 2 |
| Q04837 | Single-stranded DNA-binding protein, mitochondrial | 15.2 | 8.24 | 15 | 4 | 18 | 2 |
| Q03518 | Antigen peptide transporter 1 | 87.22 | 8.24 | 4 | 3 | 8 | 5 |
| Q03135 | Caveolin-1 | 20.34 | 5.64 | 5 | 2 | 5 | 2 |
| Q02878 | 60S ribosomal protein L6 | 32.6 | 10.59 | 12 | 5 | 13 | 5 |
| Q02543 | 60S ribosomal protein L18a | 20.76 | 10.72 | 4 | 3 | 3 | 2 |
| Q02388 | Collagen alpha-1(VII) chain | 293.6 | 5.92 | 2 | 2 | 2 | 2 |
| Q02218 | 2-oxoglutarate dehydrogenase, mitochondrial | 111.34 | 6.07 | 4 | 2 | 14 | 7 |
| Q01650 | Large neutral amino acids transporter small subunit 1 | 55.01 | 7.9 | 5 | 3 | 5 | 3 |
| Q01518 | Adenylyl cyclase-associated protein 1 | 51.77 | 8.26 | 4 | 3 | 5 | 3 |
| Q01105 | Protein SET | 33.36 | 4.22 | 17 | 9 | 4 | 2 |
| Q01082 | Spectrin beta chain, brain 1 | 274.48 | 5.39 | 9 | 6 | 9 | 6 |
| Q00839 | Heterogeneous nuclear ribonucleoprotein U | 90.45 | 5.76 | 4 | 2 | 13 | 6 |
| Q00610 | Clathrin heavy chain 1 | 191.48 | 5.48 | 45 | 25 | 72 | 36 |
| P99999 | Cytochrome c | 11.62 | 9.59 | 4 | 2 | 2 | 2 |
| P98179 | Putative RNA-binding protein 3 | 17.17 | 8.86 | 8 | 2 | 8 | 2 |
| P84077 | ADP-ribosylation factor 1 | 20.57 | 6.36 | 71 | 5 | 18 | 6 |
| P78527 | DNA-dependent protein kinase catalytic subunit | 468.09 | 6.75 | 3 | 3 | 7 | 6 |
| P78371 | T-complex protein 1 subunit beta | 57.36 | 6.02 | 25 | 8 | 25 | 8 |
| P68363 | Tubulin alpha-1B chain | 50.15 | 4.94 | 23 | 12 | 21 | 9 |
| P68133 | Actin, alpha skeletal muscle | 41.82 | 5.23 | 34 | 6 | 34 | 6 |
| P68104 | Elongation factor 1-alpha 1 | 50.14 | 9.1 | 155 | 12 | 30 | 10 |
| P68036 | Ubiquitin-conjugating enzyme E2 L3 | 17.86 | 8.68 | 2 | 2 | 2 | 2 |
| P67809 | Nuclease-sensitive element-binding protein 1 | 35.79 | 9.87 | 5 | 3 | 3 | 2 |
| P63244 | Guanine nucleotide-binding protein subunit beta-2-like 1 | 35.08 | 7.6 | 15 | 8 | 19 | 5 |
| P63241 | Eukaryotic translation initiation factor 5A-1 | 16.7 | 5.08 | 19 | 7 | 19 | 7 |
| P63220 | 40S ribosomal protein S21 | 9.11 | 8.68 | 4 | 3 | 3 | 2 |
| P63173 | 60S ribosomal protein L38 | 8..09 | 10.1 | 4 | 2 | 4 | 2 |
| P63104 | 14-3-3 protein zeta/delta | 27.75 | 4.73 | 167 | 11 | 18 | 6 |
| P63027 | Vesicle-associated membrane protein 2 | 12.53 | 7.81 | 3 | 2 | 3 | 2 |
| P63010 | AP-2 complex subunit beta | 104.42 | 5.22 | 4 | 3 | 11 | 4 |
| P62988 | Ubiquitin-60S ribosomal protein L40 | 8.56 | 6.56 | 6 | 3 | 6 | 3 |
| P62937 | Peptidyl-prolyl cis-trans isomerase A | 18.01 | 7.68 | 10 | 7 | 35 | 9 |
| P62917 | 60S ribosomal protein L8 | 27.89 | 11.04 | 8 | 4 | 8 | 4 |
| P62888 | 60S ribosomal protein L30 | 12.78 | 9.65 | 11 | 5 | 3 | 2 |
| P62879 | Guanine nucleotide-binding protein G(I)/G(S)/G(T) subunit beta-2 | 37.2 | 5.6 | 3 | 2 | 3 | 2 |
| P62873 | Guanine nucleotide-binding protein G(I)/G(S)/G(T) subunit beta-1 | 37.25 | 5.6 | 4 | 2 | 11 | 6 |
| P62851 | 40S ribosomal protein S25 | 13.74 | 10.12 | 5 | 3 | 5 | 3 |
| P62847 | 40S ribosomal protein S24 | 15.42 | 10.79 | 4 | 3 | 4 | 3 |
| P62841 | 40S ribosomal protein S15; RIG protein | 16.91 | 10.39 | 3 | 2 | 3 | 2 |
| P62829 | 60S ribosomal protein L23 | 14.87 | 10.51 | 7 | 3 | 2 | 2 |
| P62826 | GTP-binding nuclear protein Ran | 24.29 | 7.2 | 16 | 6 | 16 | 6 |
| P62820 | Ras-related protein Rab-1A | 22.55 | 5.93 | 289 | 7 | 16 | 6 |
| P62753 | 40S ribosomal protein S6 | 28.68 | 10.85 | 3 | 2 | 3 | 2 |
| P62701 | 40S ribosomal protein S4, X isoform | 29.47 | 10.16 | 18 | 7 | 10 | 4 |
| P62491 | Ras-related protein Rab-11A | 23.91 | 6.14 | 22 | 7 | 22 | 7 |
| P62424 | 60S ribosomal protein L7a | 29.86 | 10.61 | 8 | 4 | 5 | 3 |
| P62314 | Small nuclear ribonucleoprotein Sm D1 | 13.28 | 11.56 | 7 | 2 | 22 | 2 |
| P62280 | 40S ribosomal protein S11 | 18.3 | 10.31 | 2 | 2 | 2 | 2 |
| P62277 | 40S ribosomal protein S13 | 17.09 | 10.53 | 8 | 4 | 15 | 5 |

| P62269 | 40S ribosomal protein S18 | 17.59 | 10.99 | 8 | 5 | 8 | 5 |
| --- | --- | --- | --- | --- | --- | --- | --- |
| P62263 | 40S ribosomal protein S14 | 16.14 | 10.08 | 6 | 4 | 2 | 2 |
| P62258 | 14-3-3 protein epsilon | 29.17 | 4.63 | 16 | 8 | 16 | 8 |
| P62249 | 40S ribosomal protein S16 | 16.31 | 10.21 | 9 | 5 | 7 | 5 |
| P62244 | 40S ribosomal protein S15a | 14.71 | 10.14 | 67 | 5 | 67 | 5 |
| P62241 | 40S ribosomal protein S8 | 24.07 | 10.32 | 13 | 5 | 5 | 2 |
| P62136 | Serine/threonine-protein phosphatase PP1-alpha catalytic subunit | 37.38 | 5.94 | 23 | 9 | 7 | 3 |
| P62081 | 40S ribosomal protein S7 | 22.13 | 10.09 | 25 | 7 | 25 | 7 |
| P61981 | 14-3-3 protein gamma | 28.3 | 4.8 | 9 | 4 | 9 | 3 |
| P61978 | Heterogeneous nuclear ribonucleoprotein K | 50.98 | 5.39 | 5 | 4 | 5 | 4 |
| P61923 | Coatomer subunit zeta-1 | 20.2 | 4.69 | 5 | 3 | 7 | 3 |
| P61803 | Dolichyl-diphosphooligosaccharide--protein glycosyltransferase subunit DAD1 | 12.37 | 6.49 | 6 | 3 | 6 | 3 |
| P61769 | Beta-2-microglobulin | 11.73 | 6.07 | 2 | 2 | 3 | 2 |
| P61619 | Protein transport protein Sec61 subunit alpha isoform 1 | 52.13 | 8.33 | 7 | 4 | 7 | 4 |
| P61604 | 10 kDa heat shock protein, mitochondrial | 10.8 | 8.91 | 13 | 5 | 7 | 4 |
| P61353 | 60S ribosomal protein L27 | 15.8 | 10.56 | 4 | 2 | 5 | 3 |
| P61289 | Proteasome activator complex subunit 3 | 29.37 | 5.69 | 6 | 4 | 3 | 3 |
| P61247 | 40S ribosomal protein S3a | 29.81 | 9.75 | 144 | 6 | 144 | 6 |
| P61225 | Ras-related protein Rap-2b | 20.18 | 4.73 | 11 | 3 | 11 | 4 |
| P61224 | Ras-related protein Rap-1b | 20.47 | 5.65 | 53 | 4 | 53 | 4 |
| P61163 | Alpha-centractin | 42.61 | 6.19 | 5 | 3 | 2 | 2 |
| P61160 | Actin-related protein 2 | 44.76 | 6.29 | 3 | 3 | 3 | 3 |
| P61106 | Ras-related protein Rab-14 | 23.77 | 5.86 | 5 | 4 | 14 | 6 |
| P61026 | Ras-related protein Rab-10 | 22.54 | 8.58 | 10 | 2 | 10 | 2 |
| P61019 | Ras-related protein Rab-2A | 23.41 | 6.1 | 15 | 7 | 10 | 6 |
| P60953 | Cell division control protein 42 homolog | 20.93 | 6.16 | 4 | 2 | 3 | 2 |
| P60903 | Protein S100-A10; S100 calcium-binding protein A10 | 11.07 | 7.3 | 11 | 4 | 11 | 4 |
| P60900 | Proteasome subunit alpha type-6 | 27.4 | 6.34 | 4 | 2 | 2 | 2 |
| P60866 | 40S ribosomal protein S20 | 13.24 | 9.95 | 5 | 3 | 5 | 3 |
| P60709 | Actin, cytoplasmic 1 | 41.74 | 5.29 | 87 | 13 | 105 | 20 |
| P60660 | Myosin light polypeptide 6 | 16.8 | 4.56 | 12 | 4 | 12 | 4 |
| P60174 | Triosephosphate isomerase | 30.79 | 5.65 | 14 | 3 | 4 | 2 |
| P59998 | Actin-related protein 2/3 complex subunit 4 | 19.54 | 8.53 | 3 | 2 | 3 | 2 |
| P58107 | Epiplakin; 450 kDa epidermal antigen | 552.62 | 5.44 | 3 | 3 | 3 | 3 |
| P57088 | Transmembrane protein 33 | 27.85 | 9.76 | 3 | 2 | 5 | 2 |
| P56134 | ATP synthase subunit f, mitochondrial | 10.79 | 9.7 | 7 | 2 | 5 | 2 |
| P55809 | Succinyl-CoA:3-ketoacid-coenzyme A transferase 1, mitochondrial | 52.09 | 6 | 2 | 2 | 2 | 2 |
| P55084 | Trifunctional enzyme subunit beta, mitochondrial | 47.48 | 9.24 | 3 | 2 | 9 | 6 |
| P55072 | Transitional endoplasmic reticulum ATPase | 89.19 | 5.14 | 16 | 9 | 16 | 9 |
| P55060 | Exportin-2 | 110.42 | 5.51 | 12 | 5 | 6 | 3 |
| P54920 | Alpha-soluble NSF attachment protein | 33.23 | 5.23 | 4 | 3 | 4 | 3 |
| P54819 | Adenylate kinase 2, mitochondrial | 26.48 | 7.67 | 22 | 9 | 20 | 7 |
| P53675 | Clathrin heavy chain 2 | 186.9 | 5.57 | 3 | 3 | 4 | 3 |
| P53621 | Coatomer subunit alpha | 138.35 | 7.7 | 8 | 6 | 8 | 6 |
| P53618 | Coatomer subunit beta | 107.01 | 5.72 | 5 | 3 | 4 | 3 |
| P53597 | Succinyl-CoA ligase [GDP-forming] subunit alpha, mitochondrial | 32.23 | 8.77 | 5 | 2 | 5 | 2 |
| P53396 | ATP-citrate synthase | 120.84 | 6.95 | 3 | 2 | 3 | 2 |
| P52597 | Heterogeneous nuclear ribonucleoprotein F | 45.67 | 5.37 | 9 | 3 | 9 | 5 |
| P51572 | B-cell receptor-associated protein 31 | 27.86 | 8.44 | 3 | 2 | 7 | 5 |
| P51571 | Translocon-associated protein subunit delta | 16.77 | 5.5 | 10 | 4 | 10 | 4 |
| P51149 | Ras-related protein Rab-7a | 23.36 | 6.32 | 59 | 9 | 19 | 8 |
| P51148 | Ras-related protein Rab-5C | 23.48 | 8.64 | 11 | 5 | 11 | 5 |
| P50991 | T-complex protein 1 subunit delta | 57.79 | 8.13 | 19 | 9 | 3 | 2 |
| P50914 | 60S ribosomal protein L14 | 23.3 | 10.94 | 8 | 6 | 4 | 3 |
| P50454 | Serpin H1 | 44.52 | 8.81 | 11 | 4 | 11 | 4 |
| P50213 | Isocitrate dehydrogenase [NAD] subunit alpha, mitochondrial | 36.64 | 5.71 | 14 | 4 | 19 | 7 |
| P49755 | Transmembrane emp24 domain-containing protein 10 | 21.75 | 6.02 | 116 | 7 | 27 | 7 |
| P49720 | Proteasome subunit beta type-3 | 22.82 | 6.12 | 5 | 3 | 6 | 2 |
| P49411 | Elongation factor Tu, mitochondrial | 45.05 | 6.31 | 2 | 2 | 8 | 6 |
| P49368 | T-complex protein 1 subunit gamma | 60.53 | 6.1 | 10 | 6 | 10 | 6 |
| P49327 | Fatty acid synthase | 273.43 | 6.01 | 3 | 3 | 21 | 11 |
| P49257 | Protein ERGIC-53 | 54.21 | 5.75 | 7 | 4 | 4 | 3 |
| P48643 | T-complex protein 1 subunit epsilon | 59.54 | 5.44 | 12 | 4 | 4 | 2 |
| P48047 | ATP synthase subunit O, mitochondrial | 20.88 | 9.81 | 44 | 5 | 41 | 11 |
| P47914 | 60S ribosomal protein L29 | 17.62 | 11.66 | 3 | 3 | 3 | 3 |
| P46940 | Ras GTPase-activating-like protein IQGAP1 | 189.12 | 6.08 | 34 | 17 | 70 | 22 |
| P46783 | 40S ribosomal protein S10 | 18.9 | 10.15 | 4 | 2 | 3 | 2 |
| P46782 | 40S ribosomal protein S5 | 22.88 | 9.73 | 9 | 4 | 9 | 4 |
| P46781 | 40S ribosomal protein S9 | 22.46 | 10.66 | 11 | 7 | 6 | 4 |
| P46777 | 60S ribosomal protein L5 | 34.23 | 9.73 | 6 | 2 | 4 | 2 |
| P46776 | 60S ribosomal protein L27a | 16.43 | 11 | 3 | 3 | 3 | 3 |
| P45954 | Short/branched chain specific acyl-CoA dehydrogenase, mitochondrial | 43.7 | 5.72 | 5 | 2 | 5 | 4 |
| P43686 | 26S protease regulatory subunit 6B | 47.37 | 5.09 | 8 | 3 | 9 | 3 |
| P43304 | Glycerol-3-phosphate dehydrogenase, mitochondrial | 76.36 | 6.3 | 10 | 5 | 5 | 3 |
| P42892 | Endothelin-converting enzyme 1 | 87.16 | 5.61 | 3 | 2 | 3 | 2 |
| P42704 | Leucine-rich PPR motif-containing protein, mitochondrial | 151.84 | 5.53 | 6 | 5 | 84 | 34 |
| P42126 | 3,2-trans-enoyl-CoA isomerase, mitochondrial | 28.74 | 6 | 5 | 2 | 3 | 2 |
| P40939 | Trifunctional enzyme subunit alpha, mitochondrial | 79 | 8.98 | 7 | 5 | 11 | 5 |
| P40926 | Malate dehydrogenase, mitochondrial | 33 | 8.54 | 85 | 13 | 106 | 15 |
| P40616 | ADP-ribosylation factor-like protein 1 | 20.29 | 5.64 | 2 | 2 | 2 | 2 |
| P40429 | 60S ribosomal protein L13a | 23.47 | 10.94 | 5 | 4 | 3 | 2 |
| P40227 | T-complex protein 1 subunit zeta | 57.89 | 6.25 | 28 | 10 | 28 | 10 |
| P39656 | Dolichyl-diphosphooligosaccharide--protein glycosyltransferase 48 kDa subunit | 46.12 | 5.41 | 6 | 6 | 23 | 10 |
| P39023 | 60S ribosomal protein L3 | 45.98 | 10.19 | 3 | 2 | 3 | 2 |
| P39019 | 40S ribosomal protein S19 | 15.93 | 10.31 | 3 | 3 | 3 | 2 |
| P38646 | Stress-70 protein, mitochondrial | 68.76 | 5.44 | 32 | 12 | 85 | 22 |
| P38117 | Electron transfer flavoprotein subunit beta | 27.71 | 8.29 | 2 | 2 | 2 | 2 |
| P37802 | Transgelin-2; SM22-alpha homolog | 22.26 | 8.45 | 10 | 7 | 15 | 5 |
| P36957 | Dihydrolipoyllysine-residue succinyltransferase component of 2-oxoglutarate dehydrogenase complex, mitochondrial | 41.39 | 5.9 | 4 | 2 | 7 | 3 |
| P36873 | Serine/threonine-protein phosphatase PP1-gamma catalytic subunit | 36.85 | 6.14 | 5 | 2 | 5 | 2 |
| P36776 | Lon protease homolog, mitochondrial | 99.36 | 5.64 | 3 | 2 | 6 | 4 |
| P36578 | 60S ribosomal protein L4 | 47.57 | 11.07 | 7 | 5 | 7 | 5 |
| P36542 | ATP synthase subunit gamma, mitochondrial | 30.17 | 9.02 | 7 | 5 | 13 | 7 |
| P35908 | Keratin, type II cytoskeletal 2 epidermal | 65.43 | 8.07 | 2 | 2 | 2 | 2 |
| P35749 | Myosin-11 | 227.34 | 5.42 | 3 | 3 | 3 | 2 |
| P35579 | Myosin-9 | 226.4 | 5.5 | 34 | 19 | 55 | 23 |
| P35527 | Keratin, type I cytoskeletal 9 | 62.06 | 5.14 | 4 | 2 | 4 | 2 |
| P35268 | 60S ribosomal protein L22; Epstein-Barr virus small RNA-associated protein | 14.66 | 9.22 | 14 | 4 | 7 | 3 |
| P35232 | Prohibitin | 29.67 | 5.57 | 5 | 2 | 5 | 2 |
| P35221 | Catenin alpha-1 | 99.94 | 5.95 | 5 | 3 | 37 | 14 |
| P32969 | 60S ribosomal protein L9 | 21.86 | 9.96 | 19 | 4 | 11 | 4 |
| P31949 | Protein S100-A11 | 11.74 | 6.56 | 4 | 2 | 15 | 3 |
| P31947 | 14-3-3 protein sigma | 27.77 | 4.68 | 19 | 5 | 24 | 2 |
| P31946 | 14-3-3 protein beta/alpha | 28.08 | 4.76 | 17 | 7 | 17 | 7 |
| P31943 | Heterogeneous nuclear ribonucleoprotein H | 49.23 | 5.89 | 3 | 2 | 4 | 3 |
| P31942 | Heterogeneous nuclear ribonucleoprotein H3 | 36.93 | 6.37 | 2 | 2 | 2 | 2 |
| P31937 | 3-hydroxyisobutyrate dehydrogenase, mitochondrial | 31.54 | 5.54 | 2 | 2 | 5 | 2 |
| P31040 | Succinate dehydrogenase [ubiquinone] flavoprotein subunit, mitochondrial | 68.08 | 6.26 | 7 | 4 | 52 | 11 |
| P30519 | Heme oxygenase 2 | 35.9 | 5.31 | 3 | 2 | 4 | 3 |
| P30153 | Serine/threonine-protein phosphatase 2A 65 kDa regulatory subunit A alpha isoform | 65.3 | 5 | 11 | 6 | 4 | 3 |
| P30101 | Protein disulfide-isomerase A3 | 56.27 | 5.61 | 82 | 30 | 82 | 30 |
| P30084 | Enoyl-CoA hydratase, mitochondrial | 28.34 | 5.88 | 19 | 5 | 28 | 9 |
| P30050 | 60S ribosomal protein L12 | 17.82 | 9.48 | 3 | 3 | 3 | 3 |
| P30048 | Thioredoxin-dependent peroxide reductase, mitochondrial | 21.47 | 5.77 | 7 | 5 | 42 | 12 |
| P30044 | Peroxiredoxin-5, mitochondrial | 17.03 | 6.73 | 16 | 7 | 16 | 7 |
| P30040 | Endoplasmic reticulum resident protein 29 | 25.82 | 6.08 | 10 | 5 | 3 | 2 |
| P29692 | Elongation factor 1-delta | 30.99 | 4.9 | 7 | 3 | 4 | 3 |
| P29401 | Transketolase | 67.88 | 7.58 | 6 | 3 | 6 | 3 |
| P29034 | Protein S100-A2 | 11.12 | 4.68 | 5 | 3 | 14 | 4 |
| P27824 | Calnexin | 67.4 | 4.46 | 39 | 14 | 97 | 20 |
| P27797 | Calreticulin | 46.47 | 4.29 | 66 | 18 | 66 | 18 |
| P26885 | Peptidyl-prolyl cis-trans isomerase FKBP2 | 13.3 | 8.94 | 9 | 3 | 17 | 4 |
| P26641 | Elongation factor 1-gamma | 49.99 | 6.27 | 3 | 3 | 3 | 3 |
| P26599 | Polypyrimidine tract-binding protein 1 | 57.22 | 9.22 | 9 | 4 | 2 | 2 |
| P26373 | 60S ribosomal protein L13 | 24.26 | 11.65 | 7 | 4 | 7 | 4 |
| P26038 | Moesin | 67.69 | 6.09 | 22 | 10 | 22 | 10 |
| P25787 | Proteasome subunit alpha type-2 | 25.77 | 7.12 | 3 | 3 | 3 | 2 |
| P25705 | ATP synthase subunit alpha, mitochondrial | 59.21 | 8.28 | 188 | 16 | 188 | 16 |
| P25398 | 40S ribosomal protein S12 | 14.38 | 7.01 | 17 | 4 | 23 | 2 |
| P24752 | Acetyl-CoA acetyltransferase, mitochondrial | 41.39 | 8.16 | 9 | 2 | 15 | 3 |
| P24539 | ATP synthase subunit b, mitochondrial | 24.63 | 9.1 | 7 | 4 | 7 | 4 |
| P23528 | Cofilin-1 | 18.37 | 8.26 | 159 | 10 | 74 | 10 |
| P23396 | 40S ribosomal protein S3 | 26.56 | 9.68 | 21 | 10 | 10 | 6 |
| P23284 | Peptidyl-prolyl cis-trans isomerase B | 20.29 | 9.25 | 22 | 8 | 19 | 8 |
| P23229 | Integrin alpha-6 | 124.36 | 6.14 | 5 | 4 | 5 | 3 |
| P22695 | Cytochrome b-c1 complex subunit 2, mitochondrial | 48.4 | 7.74 | 4 | 3 | 4 | 3 |
| P22314 | Ubiquitin-like modifier-activating enzyme 1 | 117.72 | 5.49 | 5 | 4 | 3 | 2 |
| P21964 | Catechol O-methyltransferase | 30.04 | 5.26 | 14 | 8 | 8 | 5 |
| P21926 | CD9 antigen; p24 | 25.28 | 7.14 | 3 | 2 | 3 | 2 |
| P21912 | Succinate dehydrogenase [ubiquinone] iron-sulfur subunit, mitochondrial | 28.81 | 8.78 | 3 | 2 | 7 | 5 |
| P21333 | Filamin-A | 280.61 | 5.7 | 138 | 58 | 136 | 54 |
| P20340 | Ras-related protein Rab-6A | 23.46 | 5.42 | 7 | 4 | 6 | 5 |
| P19338 | Nucleolin | 76.48 | 4.6 | 28 | 11 | 17 | 9 |
| P18124 | 60S ribosomal protein L7 | 29.23 | 10.66 | 9 | 6 | 6 | 4 |
| P17980 | 26S protease regulatory subunit 6A | 49.2 | 5.13 | 8 | 3 | 5 | 2 |
| P17931 | Galectin-3 | 26.02 | 8.6 | 2 | 2 | 2 | 2 |
| P17301 | Integrin alpha-2 | 126.38 | 5.15 | 4 | 2 | 8 | 4 |
| P16615 | Sarcoplasmic/endoplasmic reticulum calcium ATPase 2 | 114.76 | 5.23 | 11 | 6 | 11 | 6 |
| P16435 | NADPH--cytochrome P450 reductase | 76.56 | 5.38 | 4 | 3 | 13 | 5 |

| P16144 | Integrin beta-4 | 199.44 | 6.38 | 17 | 11 | 17 | 11 |
| --- | --- | --- | --- | --- | --- | --- | --- |
| P16070 | CD44 antigen | 79.21 | 7.38 | 11 | 5 | 16 | 7 |
| P15880 | 40S ribosomal protein S2 | 31.19 | 8.38 | 11 | 5 | 13 | 7 |
| P15311 | Ezrin | 69.28 | 9.38 | 16 | 8 | 8 | 4 |
| P14625 | Endoplasmin | 90.18 | 10.38 | 303 | 31 | 138 | 29 |
| P14618 | Pyruvate kinase isozymes M1/M2 | 57.81 | 11.38 | 41 | 20 | 41 | 20 |
| P14314 | Glucosidase 2 subunit beta | 57.82 | 12.38 | 4 | 3 | 3 | 2 |
| P13804 | Electron transfer flavoprotein subunit alpha, mitochondrial | 32.97 | 13.38 | 18 | 8 | 42 | 10 |
| P13796 | Plastin-2 | 70.16 | 14.38 | 8 | 4 | 8 | 4 |
| P13726 | Tissue factor | 29.59 | 15.38 | 9 | 3 | 3 | 2 |
| P13693 | Translationally-controlled tumor protein | 19.6 | 16.38 | 3 | 2 | 3 | 2 |
| P13667 | Protein disulfide-isomerase A4 | 70.67 | 17.38 | 44 | 16 | 37 | 18 |
| P13647 | Keratin, type II cytoskeletal 5 | 62.38 | 18.38 | 6 | 2 | 6 | 2 |
| P13645 | Keratin, type I cytoskeletal 10 | 58.83 | 19.38 | 8 | 4 | 86 | 16 |
| P13639 | Elongation factor 2 | 95.21 | 20.38 | 20 | 8 | 20 | 8 |
| P13010 | X-ray repair cross-complementing protein 5 | 82.57 | 21.38 | 4 | 4 | 13 | 9 |
| P12956 | X-ray repair cross-complementing protein 6 | 69.71 | 22.38 | 16 | 10 | 16 | 10 |
| P12814 | Alpha-actinin-1 | 103.06 | 23.38 | 122 | 29 | 47 | 22 |
| P12236 | ADP/ATP translocase 3 | 32.87 | 24.38 | 2 | 2 | 6 | 5 |
| P11940 | Polyadenylate-binding protein 1 | 70.67 | 9.52 | 12 | 8 | 3 | 3 |
| P11498 | Pyruvate carboxylase, mitochondrial | 127.34 | 6.14 | 3 | 2 | 36 | 18 |
| P11279 | Lysosome-associated membrane glycoprotein 1 | 41.98 | 8.84 | 4 | 2 | 4 | 2 |
| P11233 | Ras-related protein Ral-A | 23.24 | 6.66 | 3 | 2 | 6 | 2 |
| P11142 | Heat shock cognate 71 kDa protein | 70.77 | 5.37 | 35 | 19 | 21 | 10 |
| P11021 | 78 kDa glucose-regulated protein | 70.48 | 5.01 | 230 | 32 | 230 | 32 |
| P10809 | 60 kDa heat shock protein, mitochondrial | 57.96 | 5.24 | 100 | 23 | 301 | 42 |
| P09651 | Heterogeneous nuclear ribonucleoprotein A1 | 38.62 | 9.17 | 3 | 3 | 8 | 5 |
| P09622 | Dihydrolipoyl dehydrogenase, mitochondrial | 54.17 | 6.5 | 3 | 3 | 3 | 3 |
| P09429 | High mobility group protein B1 | 24.76 | 5.6 | 10 | 5 | 2 | 2 |
| P09382 | Galectin-1 | 14.58 | 5.3 | 7 | 3 | 7 | 3 |
| P09211 | Glutathione S-transferase P | 23.32 | 5.44 | 10 | 5 | 15 | 6 |
| P08865 | 40S ribosomal protein SA | 32.72 | 4.79 | 13 | 6 | 13 | 6 |
| P08758 | Annexin A5 | 35.81 | 4.93 | 12 | 4 | 12 | 4 |
| P08574 | Cytochrome c1, heme protein, mitochondrial | 27.35 | 6.49 | 8 | 5 | 13 | 6 |
| P08238 | Heat shock protein HSP 90-beta | 83.13 | 4.96 | 34 | 13 | 45 | 16 |
| P08195 | 4F2 cell-surface antigen heavy chain | 67.99 | 4.89 | 40 | 14 | 40 | 14 |
| P08134 | Rho-related GTP-binding protein RhoC | 21.68 | 6.2 | 5 | 4 | 5 | 3 |
| P08107 | Heat shock 70 kDa protein 1A/1B | 69.92 | 5.48 | 14 | 7 | 14 | 7 |
| O00264 | Membrane-associated progesterone receptor component 1 | 21.54 | 4.54 | 7 | 2 | 10 | 3 |
| O00151 | PDZ and LIM domain protein 1 | 35.94 | 6.55 | 15 | 8 | 15 | 8 |
| O00629 | Importin subunit alpha-4 | 57.76 | 4.8 | 2 | 2 | 3 | 2 |
| O00571 | ATP-dependent RNA helicase DDX3X | 73.11 | 6.73 | 6 | 2 | 6 | 2 |
| O14579 | Coatomer subunit epsilon | 34.35 | 4.98 | 7 | 4 | 7 | 4 |
| O14980 | Exportin-1 | 123.39 | 5.71 | 4 | 2 | 4 | 3 |
| O15260 | Surfeit locus protein 4 | 30.26 | 7.81 | 5 | 2 | 6 | 3 |
| O15173 | Membrane-associated progesterone receptor component 2 | 23.82 | 4.74 | 7 | 4 | 7 | 4 |
| O43169 | Cytochrome b5 type B | 15.35 | 5.07 | 44 | 5 | 16 | 6 |
| O43399 | Tumor protein D54 | 22.24 | 5.26 | 4 | 3 | 5 | 3 |
| O43707 | Alpha-actinin-4 | 104.85 | 5.27 | 43 | 14 | 52 | 28 |
| O43852 | Calumenin | 34.96 | 4.46 | 11 | 3 | 5 | 3 |
| O60506 | Heterogeneous nuclear ribonucleoprotein Q | 69.47 | 8.68 | 9 | 4 | 5 | 4 |
| O75396 | Vesicle-trafficking protein SEC22b | 24.46 | 6.49 | 2 | 2 | 13 | 5 |
| O75390 | Citrate synthase, mitochondrial | 49.01 | 7.39 | 10 | 5 | 10 | 5 |
| O75369 | Filamin-B | 278.16 | 5.47 | 77 | 35 | 81 | 32 |
| O75489 | NADH dehydrogenase [ubiquinone] iron-sulfur protein 3, mitochondrial | 26.41 | 5.48 | 3 | 2 | 11 | 4 |
| O75964 | ATP synthase subunit g, mitochondrial | 11.3 | 9.65 | 4 | 3 | 5 | 3 |
| O75947 | ATP synthase subunit d, mitochondrial | 18.36 | 5.22 | 15 | 5 | 15 | 5 |
| O75844 | CAAX prenyl protease 1 homolog | 54.81 | 7.11 | 13 | 4 | 7 | 3 |
| O94925 | Glutaminase kidney isoform, mitochondrial | 73.4 | 7.01 | 3 | 2 | 17 | 7 |
| O95197 | Reticulon-3 | 112.5 | 4.84 | 3 | 2 | 8 | 4 |
| O95292 | Vesicle-associated membrane protein-associated protein B/C | 27.1 | 7.05 | 9 | 5 | 3 | 2 |
| O95573 | Long-chain-fatty-acid--CoA ligase 3 | 80.42 | 8.65 | 3 | 2 | 6 | 4 |
| O95881 | Thioredoxin domain-containing protein 12 | 16.43 | 5.22 | 4 | 2 | 2 | 2 |
| O95831 | Apoptosis-inducing factor 1, mitochondrial; | 55.81 | 6.86 | 2 | 2 | 2 | 2 |
| P00387 | NADH-cytochrome b5 reductase 3 | 34.1 | 7.31 | 11 | 6 | 26 | 9 |
| P00367 | Glutamate dehydrogenase 1, mitochondrial | 56.01 | 6.71 | 6 | 5 | 6 | 5 |
| P00338 | L-lactate dehydrogenase A chain. | 36.56 | 8.46 | 10 | 4 | 11 | 6 |
| P00558 | Phosphoglycerate kinase 1 | 44.48 | 8.3 | 6 | 5 | 8 | 5 |
| P00533 | Epidermal growth factor receptor | 132.01 | 6.17 | 4 | 3 | 4 | 3 |
| P00505 | Aspartate aminotransferase, mitochondrial | 44.74 | 8.98 | 13 | 7 | 24 | 12 |
| P02786 | Transferrin receptor protein 1 | 84.87 | 6.18 | 15 | 8 | 30 | 13 |
| P04406 | Glyceraldehyde-3-phosphate dehydrogenase | 35.92 | 8.58 | 503 | 24 | 425 | 34 |
| P04264 | Keratin, type II cytoskeletal 1 | 65.91 | 8.15 | 4 | 3 | 4 | 3 |
| P04181 | Ornithine aminotransferase, mitochondrial | 45.85 | 5.97 | 93 | 10 | 36 | 14 |
| P04179 | Superoxide dismutase [Mn], mitochondrial | 22.2 | 6.86 | 68 | 13 | 68 | 13 |
| P04083 | Annexin A1 | 38.58 | 6.64 | 12 | 6 | 12 | 5 |
| P04075 | Fructose-bisphosphate aldolase A | 39.29 | 8.39 | 480 | 15 | 480 | 15 |
| P07954 | Fumarate hydratase, mitochondrial | 50.08 | 6.99 | 6 | 4 | 25 | 10 |
| P07900 | Heat shock protein HSP 90-alpha | 84.53 | 4.94 | 8 | 4 | 12 | 4 |
| P07737 | Profilin-1 | 14.92 | 8.47 | 7 | 6 | 7 | 6 |
| P07602 | Proactivator polypeptide | 56.49 | 5.06 | 2 | 2 | 7 | 3 |
| P07437 | Tubulin beta chain | 49.67 | 4.78 | 13 | 7 | 13 | 7 |
| P07355 | Annexin A2 | 38.47 | 7.56 | 330 | 25 | 237 | 24 |
| P07339 | Cathepsin D | 37.85 | 5.6 | 36 | 4 | 36 | 4 |
| P07237 | Protein disulfide-isomerase | 55.29 | 4.69 | 33 | 11 | 109 | 23 |
| P06748 | Nucleophosmin | 32.58 | 4.64 | 8 | 4 | 14 | 5 |
| P06733 | Alpha-enolase | 47.04 | 6.99 | 68 | 15 | 68 | 15 |
| P06576 | ATP synthase subunit beta, mitochondrial | 51.77 | 5 | 82 | 24 | 160 | 28 |
| P05556 | Integrin beta-1 | 86.19 | 5.27 | 14 | 4 | 22 | 8 |
| P05388 | 60S acidic ribosomal protein P0 | 34.27 | 5.7 | 21 | 11 | 15 | 8 |
| P05386 | 60S acidic ribosomal protein P1 | 11.38 | 4.21 | 33 | 2 | 10 | 5 |
| P05198 | Eukaryotic translation initiation factor 2 subunit 1 | 36.11 | 5.01 | 8 | 5 | 2 | 2 |
| P05141 | ADP/ATP translocase 2 | 32.85 | 9.71 | 27 | 10 | 27 | 10 |
| P04899 | Guanine nucleotide-binding protein G(i) subunit alpha-2 | 40.32 | 5.34 | 12 | 5 | 10 | 5 |
| P04844 | Dolichyl-diphosphooligosaccharide--protein glycosyltransferase subunit 2 | 67.05 | 5.44 | 23 | 11 | 23 | 11 |
| P04843 | Dolichyl-diphosphooligosaccharide--protein glycosyltransferase subunit 1 | 66.29 | 6.03 | 12 | 6 | 42 | 15 |
| P04792 | Heat shock protein beta-1 | 22.78 | 5.98 | 3 | 2 | 3 | 2 |
| P04632 | Calpain small subunit 1 | 28.31 | 5.05 | 4 | 2 | 5 | 3 |
